# Artificial intelligence for diagnosis and Gleason grading of prostate cancer: the PANDA challenge

**DOI:** 10.1038/s41591-021-01620-2

**Published:** 2022-01-13

**Authors:** Wouter Bulten, Kimmo Kartasalo, Po-Hsuan Cameron Chen, Peter Ström, Hans Pinckaers, Kunal Nagpal, Yuannan Cai, David F. Steiner, Hester van Boven, Robert Vink, Christina Hulsbergen-van de Kaa, Jeroen van der Laak, Mahul B. Amin, Andrew J. Evans, Theodorus van der Kwast, Robert Allan, Peter A. Humphrey, Henrik Grönberg, Hemamali Samaratunga, Brett Delahunt, Toyonori Tsuzuki, Tomi Häkkinen, Lars Egevad, Maggie Demkin, Sohier Dane, Fraser Tan, Masi Valkonen, Greg S. Corrado, Lily Peng, Craig H. Mermel, Pekka Ruusuvuori, Geert Litjens, Martin Eklund, the PANDA challenge consortium, Américo Brilhante, Aslı Çakır, Xavier Farré, Katerina Geronatsiou, Vincent Molinié, Guilherme Pereira, Paromita Roy, Günter Saile, Paulo G. O. Salles, Ewout Schaafsma, Joëlle Tschui, Jorge Billoch-Lima, Emíio M. Pereira, Ming Zhou, Shujun He, Sejun Song, Qing Sun, Hiroshi Yoshihara, Taiki Yamaguchi, Kosaku Ono, Tao Shen, Jianyi Ji, Arnaud Roussel, Kairong Zhou, Tianrui Chai, Nina Weng, Dmitry Grechka, Maxim V. Shugaev, Raphael Kiminya, Vassili Kovalev, Dmitry Voynov, Valery Malyshev, Elizabeth Lapo, Manuel Campos, Noriaki Ota, Shinsuke Yamaoka, Yusuke Fujimoto, Kentaro Yoshioka, Joni Juvonen, Mikko Tukiainen, Antti Karlsson, Rui Guo, Chia-Lun Hsieh, Igor Zubarev, Habib S. T. Bukhar, Wenyuan Li, Jiayun Li, William Speier, Corey Arnold, Kyungdoc Kim, Byeonguk Bae, Yeong Won Kim, Hong-Seok Lee, Jeonghyuk Park

**Affiliations:** 1 Department of Pathology, Radboud Institute for Health Sciences, Radboud University Medical Center, Nijmegen, The Netherlands; 2 Department of Medical Epidemiology and Biostatistics, Karolinska Institutet, Stockholm, Sweden; 3 Faculty of Medicine and Health Technology, Tampere University, Tampere, Finland; 4 Google Health, Palo Alto, CA USA; 5 Department of Pathology, Antoni van Leeuwenhoek Hospital, The Netherlands Cancer Institute, Amsterdam, The Netherlands; 6 Laboratory of Pathology East Netherlands, Hengelo, The Netherlands; 7 Center for Medical Image Science and Visualization, Linköping University, Linköping, Sweden; 8 Department of Pathology and Laboratory Medicine, University of Tennessee Health Science Center, Memphis, TN USA; 9 Laboratory Medicine, Mackenzie Health, Toronto, Ontario Canada; 10 Department of Pathology, Laboratory Medicine and Pathology, University Health Network and University of Toronto, Toronto, Ontario Canada; 11 Pathology and Laboratory Medicine Service, North Florida/South Georgia Veterans Health System, Department of Pathology, Immunology and Laboratory Medicine, University of Florida, Gainesville, FL USA; 12 Department of Pathology, Yale School of Medicine, New Haven, CT USA; 13 Department of Surgery, Capio St. Göran’s Hospital, Stockholm, Sweden; 14 Aquesta Uropathology and University of Queensland, Brisbane, QLD Australia; 15 Department of Pathology and Molecular Medicine, Wellington School of Medicine and Health Sciences, University of Otago, Wellington, New Zealand; 16 Department of Surgical Pathology, School of Medicine, Aichi Medical University, Nagakute, Japan; 17 Department of Oncology and Pathology, Karolinska Institutet, Stockholm, Sweden; 18 Kaggle Inc, Mountain View, CA USA; 19 Institute of Biomedicine, Cancer Research Unit and FICAN West Cancer Centre, University of Turku and Turku University Hospital, Turku, Finland; 20 Salomão Zoppi Diagnostics/DASA, São Paulo, Brazil; 21 Pathology Department, School of Medicine, Istanbul Medipol University, Istanbul, Turkey; 22 Department of Health, Public Health Agency of Catalonia, Lleida, Spain; 23 Centre de Pathologie 68, Hopital Diaconat Mulhouse, Groupe Hospitalier de la Region Mulhouse Sud Alsace, Mulhouse, France; 24 Aix en Provence Hospital, Aix en Provence, France; 25 Histo Patologia Cirúrgica e Citologia, João Pessoa-PB, Brazil; 26 Department of Pathology, Tata Medical Center, Kolkata, India; 27 Abteilung für Histopathologie und Zytologie, Goldach, Switzerland; 28 Instituto Mário Penna, Belo Horizonte, Brazil; 29 Medics Pathologie, Bern, Switzerland; 30 HRP Labs, San Juan, PR USA; 31 Department of Pathology, Oncoclínicas group, São Paulo, São Paulo, Brazil; 32 Department of Pathology and Laboratory Medicine, Tufts Medical Center, Boston, MA USA; 33 Texas A&M University, College Station, TX USA; 34 Sungnam-ci, Republic of Korea; 35 Department of Health Informatics, Kyoto University, Kyoto, Japan; 36 Preferred Networks Inc., Tokyo, Japan; 37 Nowcast Inc., Tokyo, Japan; 38 School of Biological Science and Medical Engineering, Southeast University, Nanjing, China; 39 CTAccel Ltd., ShenZhen, China; 40 Jumio Corp., Montréal, Canada; 41 ELEME Inc., Shanghai, China; 42 School of Computer Science and Engineering, Beihang University, Beijing, China; 43 DTU Compute, Technical University of Denmark, Lyngby, Denmark; 44 Moscow, Russia; 45 Department of Materials Science and Engineering, University of Virginia, Charlottesville, VA USA; 46 Nairobi, Kenya; 47 Biomedical Image Analysis Department, The United Institute of Informatics Problems, Minsk, Belarus; 48 Madrid, Spain; 49 Systems Research & Development Center, Technology Bureau, NS Solutions Corp., Kanagawa, Japan; 50 Rist Inc., Tokyo, Japan; 51 Wireless System Lab., Toshiba Corp., Kawasaki, Japan; 52 Silo AI, Turku, Finland; 53 University of Turku, Turku, Finland; 54 University of Michigan, Ann Arbor, MI USA; 55 Taipei City, Taiwan; 56 Tula, Russia; 57 Janelia Research Campus, Ashburn, VA USA; 58 Computational Diagnostics Lab, University of California, Los Angeles, Los Angeles, CA USA; 59 VUNO Inc., Seoul, Republic of Korea

**Keywords:** Machine learning, Prostate cancer, Medical imaging

## Abstract

Artificial intelligence (AI) has shown promise for diagnosing prostate cancer in biopsies. However, results have been limited to individual studies, lacking validation in multinational settings. Competitions have been shown to be accelerators for medical imaging innovations, but their impact is hindered by lack of reproducibility and independent validation. With this in mind, we organized the PANDA challenge—the largest histopathology competition to date, joined by 1,290 developers—to catalyze development of reproducible AI algorithms for Gleason grading using 10,616 digitized prostate biopsies. We validated that a diverse set of submitted algorithms reached pathologist-level performance on independent cross-continental cohorts, fully blinded to the algorithm developers. On United States and European external validation sets, the algorithms achieved agreements of 0.862 (quadratically weighted κ, 95% confidence interval (CI), 0.840–0.884) and 0.868 (95% CI, 0.835–0.900) with expert uropathologists. Successful generalization across different patient populations, laboratories and reference standards, achieved by a variety of algorithmic approaches, warrants evaluating AI-based Gleason grading in prospective clinical trials.

## Abstract (web summary)

Through a community-driven competition, the PANDA challenge provides a curated diverse dataset and a catalog of models for prostate cancer pathology, and represents a blueprint for evaluating AI algorithms in digital pathology.

## Main

Gleason grading^1^ of biopsies yields important prognostic information for prostate cancer patients and is a key element for treatment planning^2^. Pathologists characterize tumors into different Gleason growth patterns based on the histological architecture of the tumor tissue. Based on the distribution of Gleason patterns, biopsy specimens are categorized into one of five groups, commonly referred to as International Society of Urological Pathology (ISUP) grade groups, ISUP grade, Gleason grade groups or simply grade groups (GGs)^3–6^. This assessment is inherently subjective with considerable inter- and intrapathologist variability^7,8^, leading to both undergrading and overgrading of prostate cancer^8–10^.

AI algorithms have shown promise for grading prostate cancer^11,12^, specifically in prostatectomy samples^13,14^ and biopsies^15–18^, and by assisting pathologists in the microscopic reviews^19,20^. However, AI algorithms are susceptible to various biases in their development and validation^21,22^. This can result in algorithms that perform poorly outside the cohorts used for their development. Moreover, shortcomings in validating the algorithms’ performance on additional cohorts may lead to such deficiencies in generalization going unnoticed^23,24^. Algorithms are also often developed and validated in a siloed manner: the same researchers who develop the algorithms also validate them. This leads to risks of introducing positive bias, because the developing researchers have control over, for example, establishing the validation cohorts and selecting the pathologists providing the reference standard. There has yet to be an independent evaluation of algorithms for prostate cancer diagnosis and grading to assess whether they generalize across different patient populations, pathology labs, digital pathology scanner providers and reference standards derived from intercontinental panels of uropathologists. This represents a key barrier to implementation of algorithms in clinical practice.

AI competitions have been an effective approach to crowd source the development of performant algorithms^25–27^. Despite their effectiveness in facilitating innovation, competitions still tend to suffer from a set of limitations. Validation of the resulting algorithms has typically not been performed independently of the algorithm developers. In a competitive setup, the incentive for conscious or subconscious introduction of positive bias by the developers is arguably further increased, and a lack of independent validation also means that the technical reproducibility of the proposed solutions is not verified. Moreover, competitions have typically not been followed up by validation of the algorithms on additional international cohorts, casting doubt on whether the resulting solutions possess the generalization capability to truly answer the underlying clinical problem, as opposed to being fine-tuned for a particular competition design and dataset^28^.

Through the present study, we aimed to advance the methodology for the design and evaluation of medical imaging AI innovations to develop and rigorously validate the next generation of algorithms for prostate cancer diagnostics. We organized a global AI competition, the Prostate cANcer graDe Assessment (PANDA) challenge, by compiling and publicly releasing a European (EU) cohort for AI development, the largest publicly available dataset of prostate biopsies to date. Second, we fully reproduced top-performing algorithms and externally validated their generalization to independent US and EU cohorts and compared them with the reviews of pathologists. The competition setup isolated the developers from the independent evaluation of the algorithms’ performance, minimizing the potential for information leakage and offering a true assessment of the diagnostic power of these techniques. Taken together, we show how the combination of AI and innovative study designs, together with prespecified and rigorous validation across diverse cohorts, can be utilized to solve challenging and important medical problems.

## Results

### Characteristics of the datasets

**Extended Data Fig. 1:**
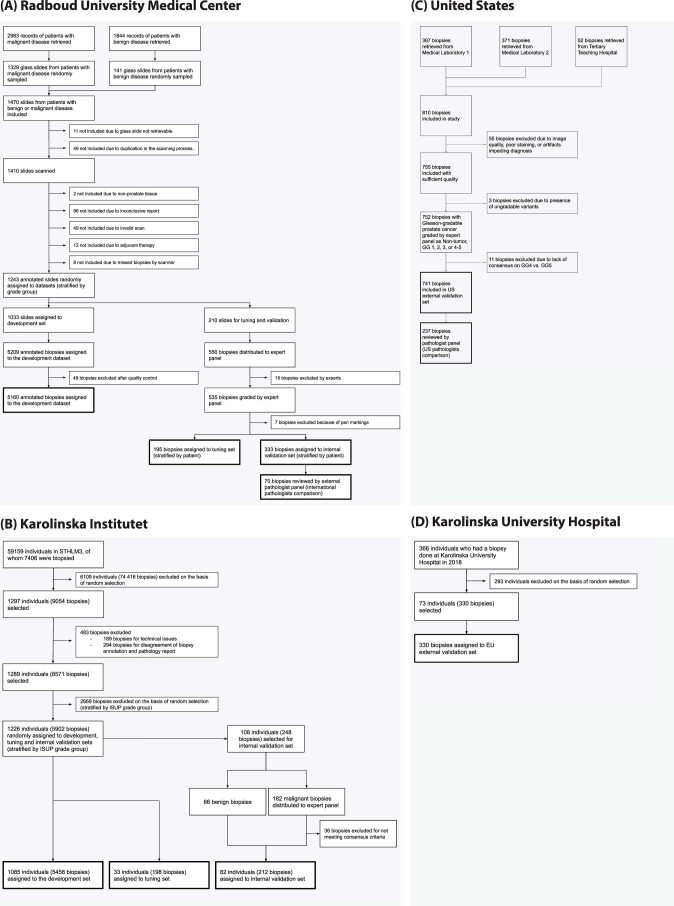
Flow charts of inclusion and exclusion for the various datasets. (**a**) Data originating from Radboud University Medical Center (development, tuning and internal validation sets, and international pathologist comparison), (**b**) Data originating from Karolinska Institutet (development, tuning and internal validation sets), (**c**) Data originating from the United States (US external validation set, and US pathologist comparison), (**d**) Data originating from Karolinska University Hospital (EU external validation set).

In total, 12,625 whole-slide images (WSIs) of prostate biopsies were retrospectively collected from 6 different sites for algorithm development, tuning and independent validation (Table 1, Extended Data Fig. 1 and Supplementary Tables 7 and 8). Of these, 10,616 biopsies were available for model development (the development set), 393 for performance evaluation during the competition phase (the tuning set), 545 as the internal validation set in the postcompetition phase and 1,071 for external validation.

**Table 1:** Data characteristics of the development set, tuning set, internal validation set and the two external validation sets

	EU development set	EU development set	EU tuning set	EU tuning set	EU internal validation set	EU internal validation set	US external validation set	EU external validation set	Total
Source	Radboud University Medical Center Netherlands	Karolinska Institutet Sweden	Radboud University Medical Center Netherlands	Karolinska Institutet Sweden	Radboud University Medical Center Netherlands	Karolinska Institutet Sweden	Medical Laboratories, CA/UT, USA; tertiary teaching hospital, CA, USA	Karolinska University Hospital Sweden	–
No. of sites	1	1	1	1	1	1	3	1	6
No. of cases	1,028	1,085	72	33	129	82	741	330	3,500
No. of biopsies	5,160	5,456	195	198	333	212	741	330	12,625
Nontumor	967 (19)	1,925 (35)	95 (49)	58 (29)	155 (47)	66 (31)	254 (34)	108 (33)	3,628 (29)
Tumor-containing (ISUP GG breakdown below)	4,193 (81)	3,531 (65)	100 (51)	140 (71)	178 (53)	146 (69)	487 (66)	222 (67)	8,997 (71)
GG 1	852 (17)	1,814 (33)	24 (12)	48 (24)	48 (14)	53 (25)	247 (33)	65 (20)	3,151 (25)
GG 2	675 (13)	668 (12)	15 (8)	32 (16)	35 (11)	34 (16)	122 (16)	63 (19)	1,644 (13)
GG 3	925 (18)	317 (6)	15 (8)	14 (7)	38 (11)	16 (8)	70 (9)	49 (15)	1,444 (11)
GG 4	768 (15)	481 (9)	19 (10)	30 (15)	16 (5)	22 (10)	21 (3)	19 (6)	1,376 (11)
GG 5	973 (19)	251 (5)	27 (14)	16 (8)	41 (12)	21 (10)	27 (4)	26 (8)	1,382 (11)
No. of cases with general pathologist reviews	–	–	–	**–**	70	**–**	237	**–**	307
No. of pathologist reviews	**–**	**–**	**–**	**–**	910	**–**	4,740	**–**	5,650

The values in parentheses give the percentage. The development set was available to competition teams for algorithm development, and the tuning set for limited algorithm evaluation during the competition. All validation sets were fully independent and blinded to the algorithm developers. Additional details on reference standard protocol can be found in Supplementary Methods 2 and 3.

Cases for development, tuning and internal validation originated from Radboud University Medical Center, Nijmegen, the Netherlands and Karolinska Institutet, Stockholm, Sweden (Extended Data Fig. 1 and Supplementary Methods 1, 2 and 3). The external validation data consisted of a US and an EU set. The US set contained 741 cases and was obtained from two independent medical laboratories and a tertiary teaching hospital. The EU external validation set contained 330 cases and was obtained from the Karolinska University Hospital, Stockholm, Sweden. The histological preparation and scanning of the external validation samples were performed by different laboratories to those responsible for the development, tuning and internal validation data.

### Reference standards of the datasets

The reference standard for the Dutch part of the training set was determined based on the pathology reports from routine clinical practice. For the Swedish part of the training set, the reference standard was set by one uropathologist (L.E.) following routine clinical workflow. The reference standard for the Dutch part of the internal validation set was determined through consensus of three uropathologists (C.H.v.d.K., R.V. and H.v.B.) from two institutions with 18–28 years of clinical experience after residency (mean of 22 years). For the Swedish subset, four uropathologists (L.E., B.D., H.S. and T.T.) from four institutions, all with >25 years of clinical experience after residency, set the reference standard.

For the US external validation set, the reference standard was set by a panel of six US or Canadian uropathologists (M.A., A.E., T.v.d.K., M.Z., R.A. and P.H.) from six institutions with 18–34 years of clinical experience after residency (mean of 25 years). Each specimen was first reviewed by two uropathologists from the panel. A third uropathologist reviewed discordant cases to arrive at a majority opinion. For this external dataset, immunohistochemistry was available to aid in tumor identification. The EU external validation set was reviewed by a single uropathologist (L.E.). For details on the uropathologist review protocol, see Supplementary Methods 2. On validation sets, the pathologists who contributed to the reference standards showed high pairwise agreement (0.926 on a subset of the EU internal validation set and 0.907 on the US external validation set, Supplementary Table 6). To ensure consistency across different reference standards, we additionally investigated the agreement between reference standards across continents (Supplementary Table 9). We found high agreement between pathologists across the regions when EU uropathologists reviewed US data and vice versa. Moreover, majority votes of the panels were highly consistent with the reference standard of the other region (quadratically weighted κ 0.939 and 0.943 for, respectively, the EU and US pathologists, Supplementary Table 9).

### Overview of the competition

**Fig. 1:**
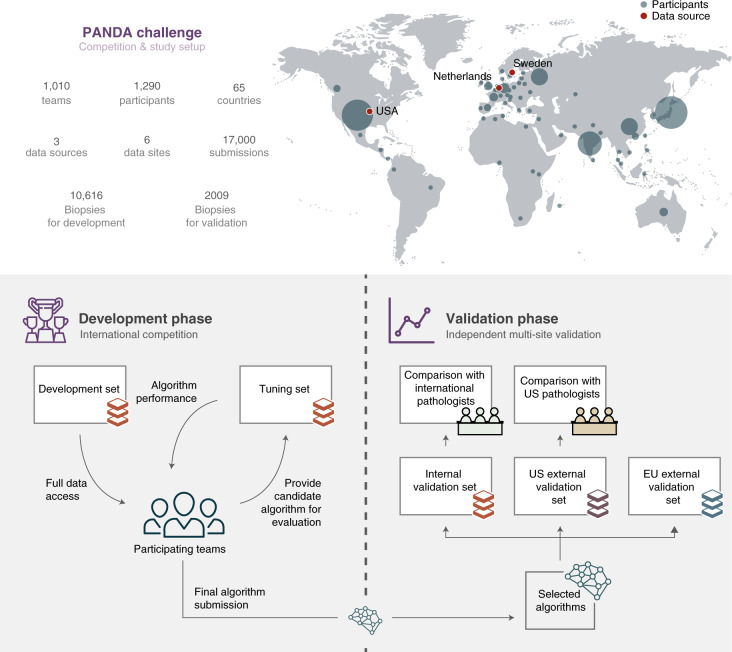
Overview of the PANDA challenge and study setup. The global competition attracted participants from 65 countries (top: size of the circle for each country illustrates the number of participants). The study was split into two phases. First, in the development phase (bottom left), teams competed in building the best-performing Gleason grading algorithm, having full access to a development set for algorithm training and limited access to a tuning set for estimating algorithm performance. In the validation phase (bottom right), a selection of algorithms was independently evaluated on internal and external datasets against reference grading obtained through consensus across expert uropathologist panels, and compared with groups of international and US general pathologists on subsets of the data.

**Fig. 2:**
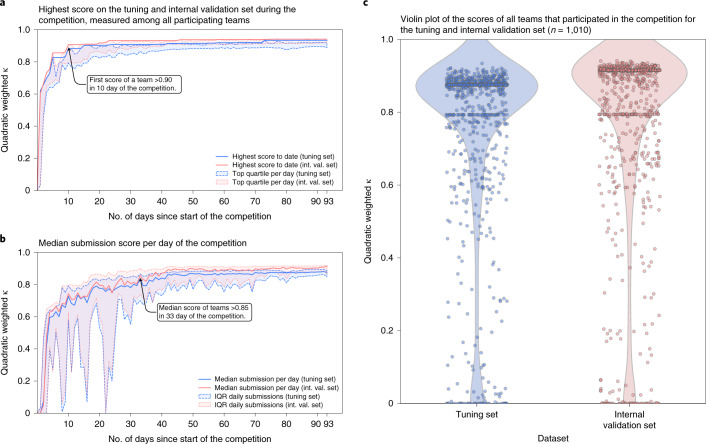
Progression of algorithms’ performances throughout the competition. During the competition, teams could submit their algorithm for evaluation on the tuning set, after which they received their score. At the same time, algorithms were evaluated on the internal validation set, without disclosing these results to the participating teams. **a**,**b**, The development of the top score obtained by any team (**a**) and the median score over all daily submissions (**b**) throughout the timeline of the competition showing the rapid improvement of the algorithms. **c**, A large fraction of teams reached high scores in the range 0.80–0.90, and retained their performance on the internal validation set. Source data

The study design of the PANDA challenge was preregistered^29^ and consisted of a competition and a validation phase. The competition was open to participants from 21 April until 23 July 2020 and was hosted on the Kaggle platform (Supplementary Methods 5). During the competition phase, 1,010 teams, consisting of 1,290 developers from 65 countries, participated and submitted at least one algorithm (Fig. 1). Throughout the competition, teams could request evaluations of their algorithm on the tuning set (Supplementary Methods 2). The algorithms were then simultaneously blindly validated on the internal validation set (Fig. 2). All teams combined submitted 34,262 versions of their algorithms, resulting in a total of 32,137,756 predictions made by the algorithms.

The first team to achieve an agreement with the uropathologists of >0.90 (quadratically weighted Cohen’s κ) on the internal validation set already occurred within the first 10 days of the competition (Fig. 2). In the 33rd day of the competition, the median performance of all teams exceeded a score of 0.85.

### Overview of evaluated algorithms

After the competition, teams were invited to join the PANDA consortium. Of all teams, 33 submitted a proposal to join the validation phase of the study. From these, the competition organizers selected 15 teams based on their algorithm’s performance on the internal validation set and method description (Supplementary Methods 6). Among the 10 highest ranking teams in the competition, 8 submitted a proposal and were accepted to join the consortium. A further seven teams in the consortium all ranked within the competition’s top 30.

All selected algorithms made use of deep learning-based methods^30,31^. Many of the solutions demonstrated the feasibility of end-to-end training using case-level information only^32^, that is, using the International Society of Urological Pathology (ISUP) GG of a specimen as the target label for an entire WSI. Most leading teams, including the winner of the competition, adopted an approach in which a sample of smaller images, or patches, is first extracted from the WSI. The patches are then fed to a convolutional neural network, the resulting feature responses are concatenated and the final classification layers of the network are applied to these features. This allows training a single model end-to-end in a computationally efficient fashion to directly predict the ISUP GG of a WSI. Such weakly supervised approaches do not require detailed pixel-level annotations as often used in fully supervised training.

Another algorithmic feature adopted by several top-performing teams was to apply automated label cleaning, where samples considered as erroneously graded by the pathologists were either excluded from training or relabeled. Several teams indicated the label noise associated with the subjective grading assigned by pathologists as a key problem, and tackled it by algorithms that detect samples where the reference standard deviates considerably from the predictions of the model. Label denoising was then typically applied iteratively to refine the labels more aggressively as the model’s performance improved during training.

A third key feature shared by all teams of the PANDA consortium was the use of ensembles consisting of diverse models, featuring, for example, different data preprocessing approaches or different neural network architectures. Despite the relative diversity in these algorithmic details, by averaging the predictions of the models constituting the ensembles, most teams achieved comparable overall performance.

For a summary and details on the individual algorithms see Supplementary Methods 7 and Supplementary algorithm descriptions. Most of the evaluated algorithms are available freely for research use (please see Supplementary algorithm descriptions for further details).

### Classification performance in the internal validation dataset

**Fig. 3:**
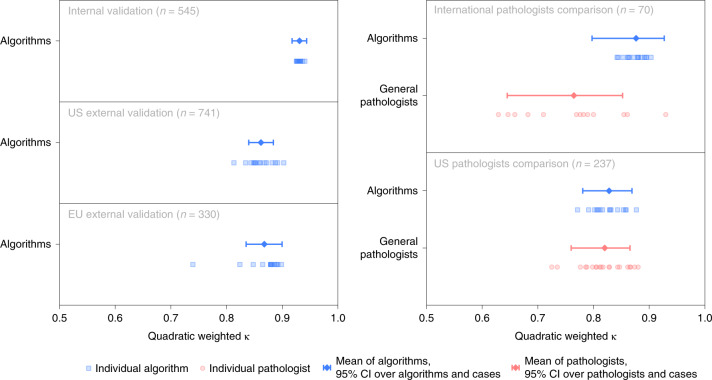
Algorithm agreement with reference standards and comparison to pathologists. Algorithms’ agreement (quadratically weighted κ) with reference standards established by uropathologists shown for the internal and external validation sets (left). On subsets of the internal and US external validation sets, agreement of general pathologists with the reference standards is additionally shown for comparison (right).

**Fig. 4:**
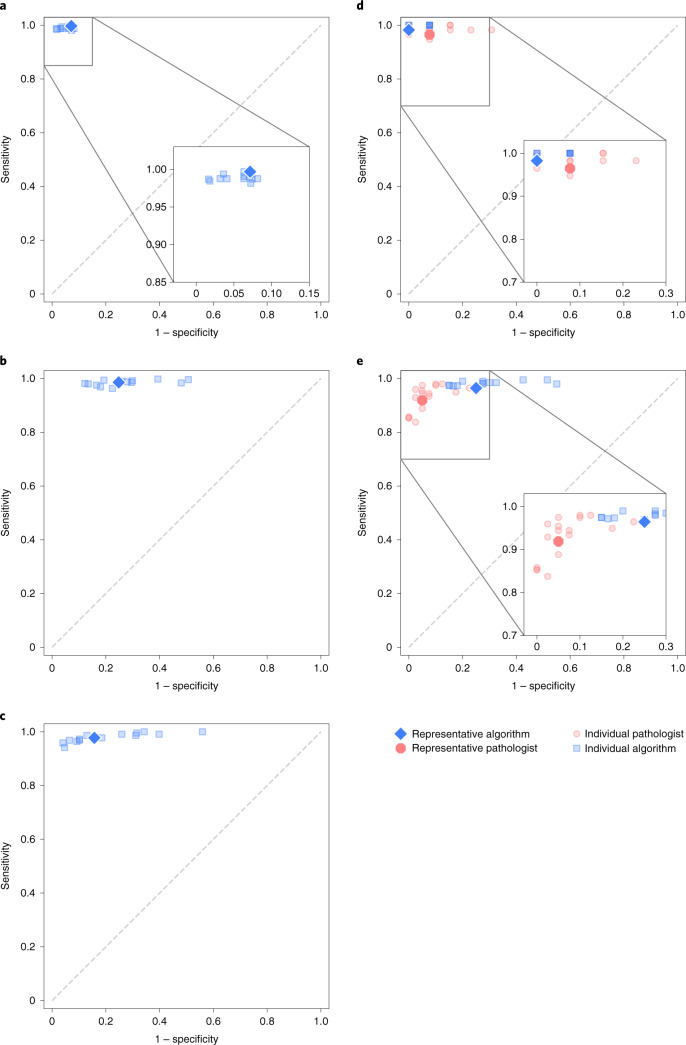
Algorithm performance in detecting prostate tumors on validation sets and comparison to pathologists. **a,b,c**, The sensitivity and specificity of the algorithms relative to reference standards established by uropathologists shown for the internal (**a**) and external validation sets (**b**, **c**). **b**, US external validation. **c**, EU external validation. **d**,**e**, On subsets of the internal and US external validation sets, the sensitivity and specificity of general pathologists are also shown for comparison. **d**, International pathologists' comparison. **e**, US pathologists' comparison.

**Extended Data Fig. 2:**
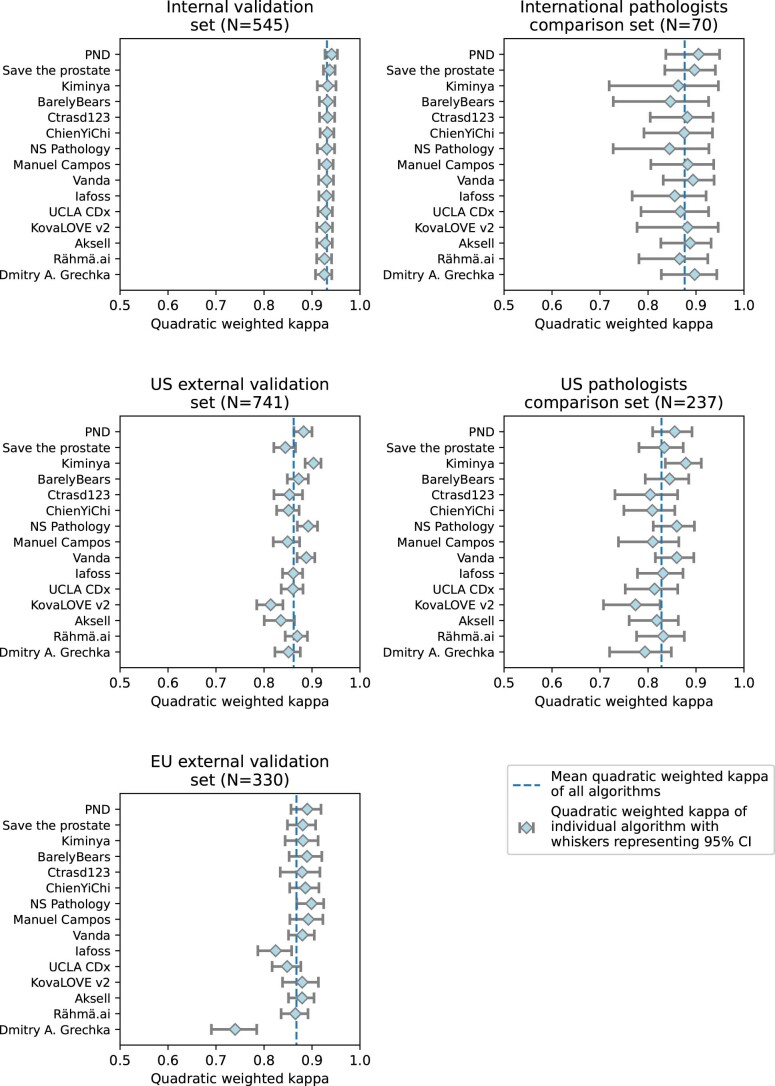
Individual algorithms’ agreement with the reference standard for the validation sets. Concordance with ISUP GG of the reference standard (Cohen’s quadratically weighted kappa with 95% CI over cases) is shown for each algorithm on each validation set. The dashed line indicates the mean of all teams on the validation set in question.

**Extended Data Fig. 3:**
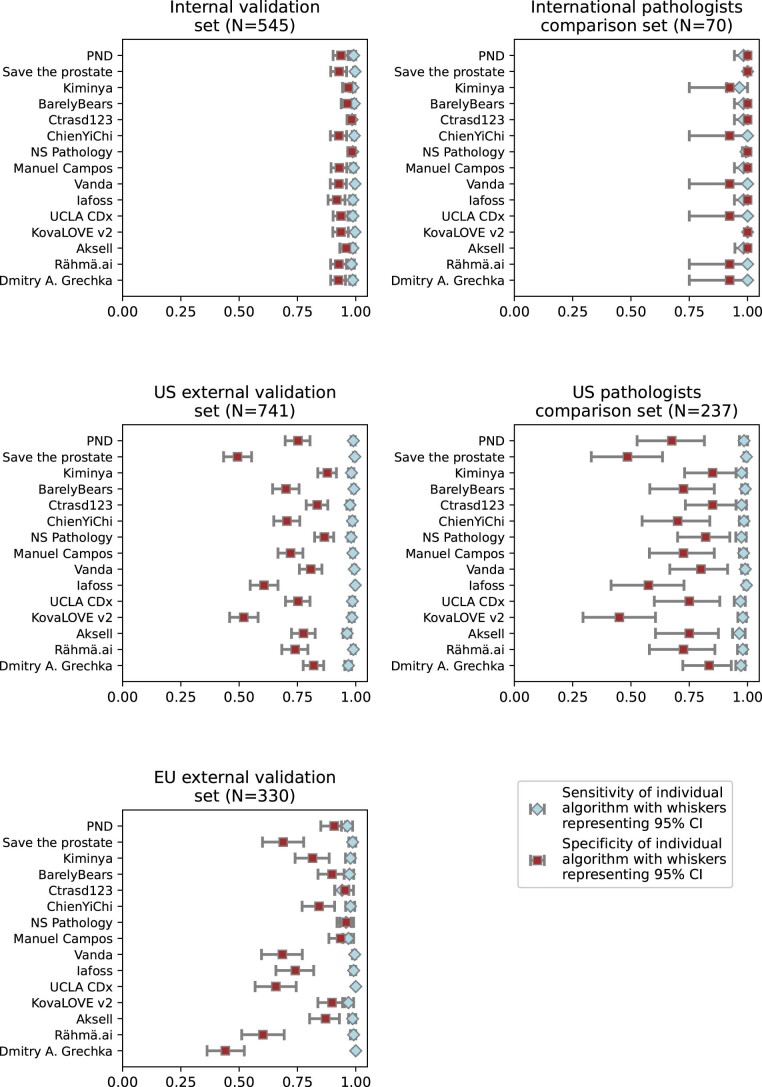
Individual algorithms’ Sensitivity and specificity for the validation sets. Performance in detecting biopsies containing cancer (sensitity and specificity with 95% CI over cases) is shown for each algorithm on each validation set.

**Extended Data Fig. 4:**
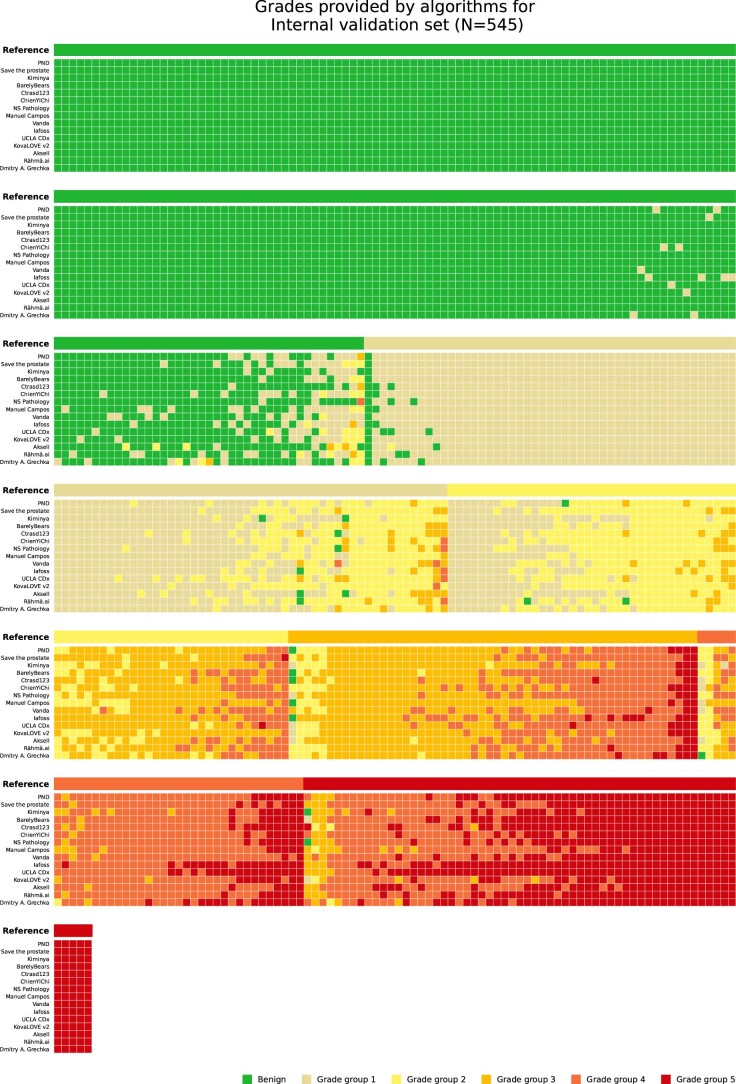
Visualization of grade assignment by algorithms for the internal validation set. Cases are ordered by the reference ISUP grade group and average grade group of the AI cohort.

In the validation phase, all selected algorithms were fully reproduced on two separate computing platforms. The average agreement of the selected algorithms with the uropathologists was high with a quadratically weighted κ of 0.931 (95% CI, 0.918–0.944, Fig. 3). Algorithms showed high sensitivity for tumor detection, with the representative algorithm (selected based on median balanced accuracy, see Statistical analysis) achieving a sensitivity of 99.7% (95% CI of all algorithms, 98.1–99.7, Fig. 4) and a specificity of 92.9% (95% CI of all algorithms, 91.9–96.7). The classification performances of the individual algorithms are presented in Extended Data Figs. 2–4 and Supplementary Tables 2 and 3.

### Classification performance in the external validation datasets

**Extended Data Fig. 5:**
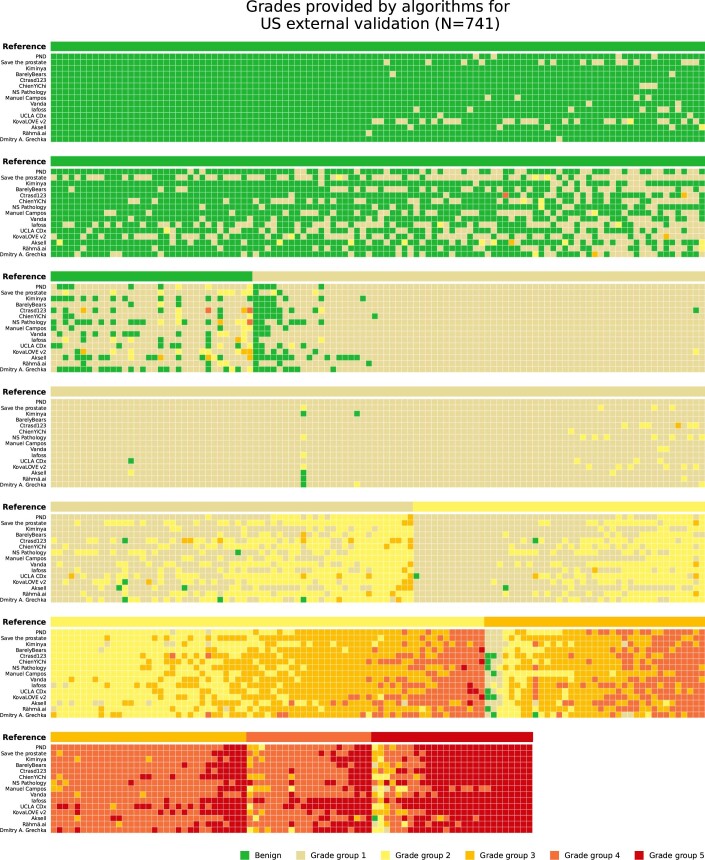
Visualization of grade assignment by algorithms for the US external validation set. Cases are ordered by the reference ISUP grade group and average grade group of the AI cohort.

**Extended Data Fig. 6:**
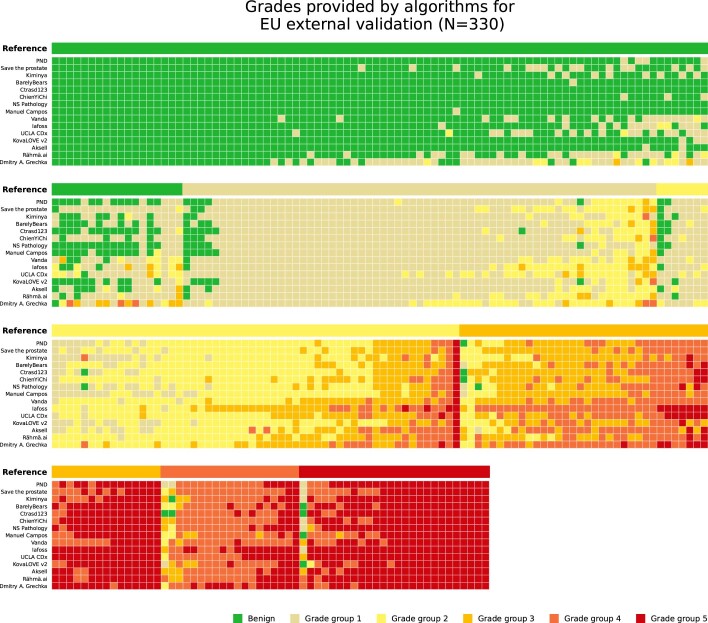
Visualization of grade assignment by algorithms for the EU external validation set. Cases are ordered by the reference ISUP grade group and average grade group of the AI cohort.

The algorithms were independently evaluated on the two external validation sets. The agreements with the reference standards were high with a quadratically weighted κ of 0.862 (95% CI, 0.840–0.884) and 0.868 (95% CI, 0.835–0.900) for the US and EU external validation sets, respectively. The main algorithm error mode was overdiagnosing of benign cases as ISUP GG 1 cancer (Extended Data Figs. 5 and 6).

The representative algorithm identified cases with tumor in the external validation sets, with sensitivities of 98.6% (95% CI of all algorithms, 97.6–99.3) and 97.7% (95% CI of all algorithms, 96.2–99.2) for the US and EU sets, respectively. In comparison to the internal validation set, the algorithms misclassified more benign cases as malignant, resulting in specificities of 75.2% (95% CI of all algorithms, 66.8–80.0) and 84.3% (95% CI of all algorithms, 70.5–87.9) for the representative algorithm.

### Classification performance compared with pathologists

To compare algorithms’ performances with those of general pathologists, we obtained reviews from two panels of pathologists on subsets of the internal and US external validation sets. For the Dutch part of the internal validation set, 13 pathologists from 8 countries (7 from Europe and 6 outside of Europe) reviewed 70 cases. For the US external validation set, 20 US board-certified pathologists reviewed 237 cases. For details on the pathologist review protocol, see Supplementary Methods 3.

**Fig. 5:**
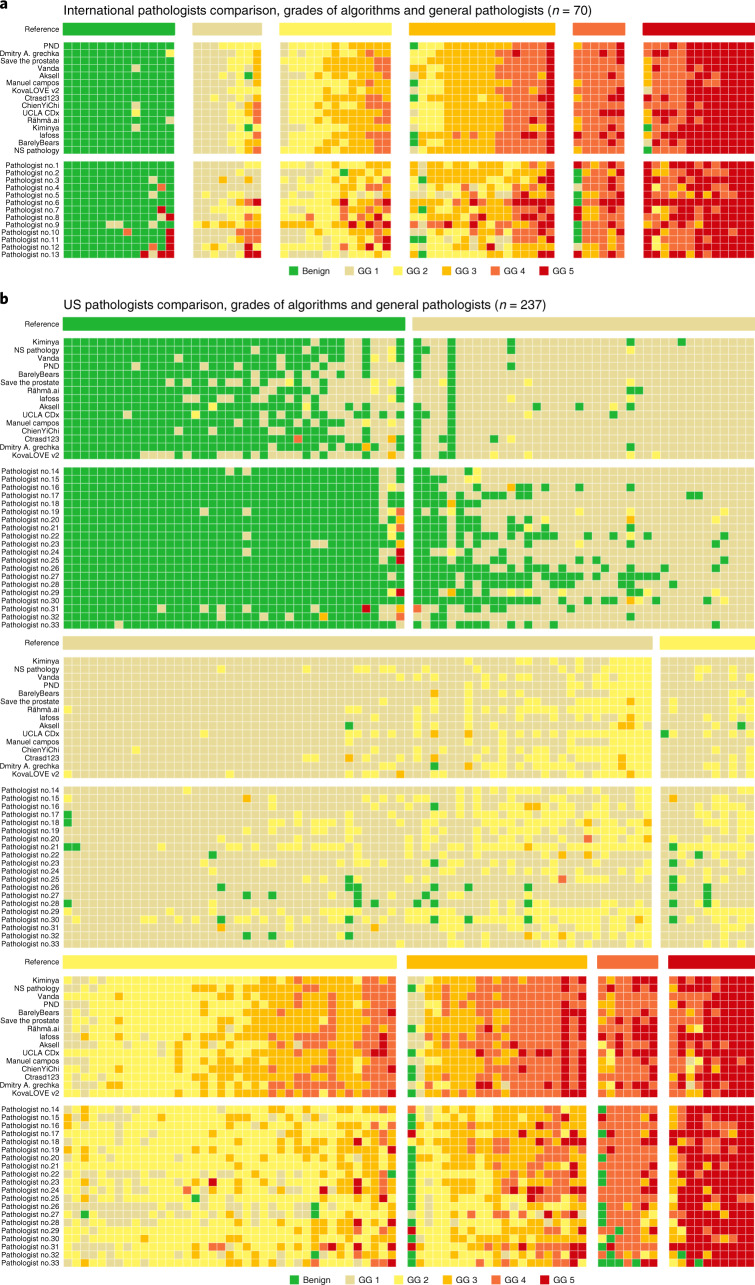
ISUP GG assignment by algorithms and pathologists. **a**,**b**, Algorithms compared with international general pathologists on a subset of the internal validation set (**a**) and US general pathologists on a subset of the US external validation set (**b**). Cases are ordered primarily by the reference ISUP GG and secondarily by the average GG of the algorithms and pathologists. Algorithms and pathologists are ordered by their agreement (quadratically weighted κ) with the reference standard on the respective sets. The comparison between pathologists and algorithms gives insight into the difference in their operating points and for which GGs most miscalls are made. The algorithms are less likely to miss a biopsy containing cancer, but at the same time more likely to overgrade benign cases.

The algorithms scored significantly (*P* < 0.001) higher in agreement with the uropathologists (0.876, 95% CI, 0.797–0.927; Fig. 3) than the international general pathologists did (0.765, 95% CI, 0.645–0.852) on the 70 cases from the Dutch part of the internal validations set. The representative algorithm had higher sensitivity for tumor (98.2%, 95% CI of all algorithms 97.4–100.0) than the representative pathologist (96.5%, 95% CI of all pathologists 95.4–100.0) and higher specificity (100.0%, 95% CI of all algorithms 90.6–100.0, versus 92.3%, 95% CI of all pathologists 77.8–97.8). On average, the algorithms missed 1.0% of cancers, whereas the pathologists missed 1.8%. Differences in grade assignments between the algorithms and pathologists are visualized in Fig. 5.

On the subset of the US external validation set with pathologist reviews, the algorithms exhibited a similar level of agreement with the uropathologists as the US general pathologists did (0.828, 95% CI, 0.781–0.869 versus 0.820, 95% CI, 0.760–0.865; *P* = 0.53). The representative algorithm had higher sensitivity for tumor (96.4%, 95% CI of all algorithms, 96.6–99.5) than the representative pathologist (91.9%, 95% CI of all pathologists, 89.3–95.5) but lower specificity (75.0%, 95% CI of all algorithms, 61.2–82.7 versus 95.0%, 95% CI of all pathologists, 87.4–98.1). On average, the algorithms missed 1.9% of cancers, whereas the pathologists missed 7.3%.

## Discussion

AI has shown promise for diagnosis and grading of prostate cancer, but these results have been restricted to siloed studies with limited proof for generalization across diverse multinational cohorts, representing one of the central barriers to implementation of AI algorithms in clinical practice. The objective of the present study was to overcome these critical issues. First, we aimed to facilitate community-driven development of AI algorithms for cancer detection and grading on prostate biopsies. Second, we sought to transcend isolated assessment of the diagnostic performance of individual AI solutions by focusing on reproducibility and fully blinded validation of a diverse group of algorithms on intercontinental and multinational cohorts.

**Extended Data Fig. 7:**
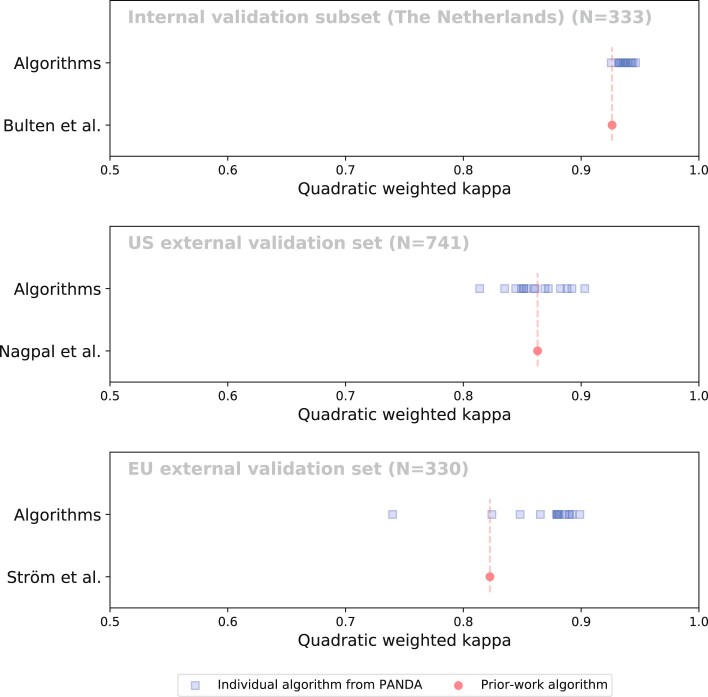
Comparison of challenge algorithms to prior work. The performance of the teams’ algorithms was computed on validation (sub)sets of earlier work. For each validation set, we additionally show the performance of the original algorithm. Source data

The resulting PANDA challenge was, to the best of our knowledge, the largest competition in pathology organized to date, in terms of both the number of participants and the size of the datasets, and the first study to analyze a variety of AI algorithms for computational pathology on this scale^33^. The datasets included variability in biopsy sampling procedure, specimen preparation process and whole-slide scanning equipment, and had different and multinational sets of pathologists contributing to the reference standard of the validation sets. Our main finding was that AI algorithms obtained from a competition setup could successfully detect and grade tumors, reaching pathologist-level concordance with expert reference standards. We further compared the algorithms with previously published results (Supplementary Table 5 and Extended Data Fig. 7)^15–17^. The algorithms outperformed earlier works on subsets of the EU validation sets. On the US external validation set, the algorithms reached similar performance without any fine-tuning, demonstrating a successful generalization to an unseen independent validation set and beyond any current state of the art. Last, groups of international and US pathologists also reviewed subsets of the internal and external validation datasets. The algorithms had a concordance with the reference standard that was similar to or higher than that of these pathologists.

In the external validation sets, the main algorithm error mode was overdiagnosing benign cases as ISUP GG 1. This is probably due to the data distribution shift between training data and external validation data^34^, in combination with the study design of independent validation, where the teams did not have any access to the validation sets, potentially leading to suboptimal selection of operating thresholds based only on the tuning set. We observed this in the US external validation set (Fig. 4), where the algorithms appear to be shifted toward higher sensitivity but lower specificity compared with the general pathologists. A potential solution to address the natural data distribution shift is to calibrate the models’ predictions using sampled data from the target sites. In addition, we showed high consistency between reference standards (Supplementary Table 9), adding additional proof that the performance drop was not caused by a difference in grading characteristics.

In the US external validation set, tumor identification was confirmed by immunohistochemistry, supporting the finding that the algorithms missed fewer cancers than the pathologists. This higher sensitivity shows promise for reducing pathologist workload by automated identification and exclusion of most benign biopsies from review. Analysis of an ensemble constructed from the algorithms suggests that combining existing algorithms could improve specificity (Supplementary Table 4).

Further analysis of the grade assignments by the algorithms and general pathologists showed that the algorithms tended to assign higher grades than the pathologists (Fig. 5). For example, in the US external validation set, algorithms overgraded a substantial portion of ISUP GG 3 cases as GG 4. The general pathologists, in contrast, tended to undergrade cases, most notably in the high-grade cases. These differences suggest that general pathologists supported by AI could reach higher agreements with uropathologists, potentially alleviating some of the rater variability associated with Gleason grading^19,20^. It should be noted that the algorithms’ operating points were selected solely based on the EU tuning set. For clinical usage, the operating points can be adjusted based on the needs and the intended use cases. For example, for a prescreening use case aimed to reduce pathologist workload, one could select an operating point favoring high sensitivity to minimize false negatives. Alternatively, if AI was used as a stand-alone tool, increasing the algorithms’ specificity to tumors, while retaining a high sensitivity, could be an important prerequisite for clinical implementation to prevent overdiagnosis.

We aimed to lower the entry barrier to medical AI development by providing access to a large, curated dataset, typically attainable only through large research consortia, and by organizing this competition to facilitate joint development with experience sharing among the teams. The results show that the publication of such datasets can lead to rapid development of high-performing AI algorithms. Dissemination and fast iteration of new ideas resulted in the first team achieving pathologist-level performance in the first 10 days of the challenge (Fig. 2). These results show the important role data play in the development of medical AI algorithms, given the short lead-time of top-performing solutions by various teams. At the same time, often raised criticisms of medical AI challenges are the lack of detailed reporting, and limited interpretation and reproducibility of results^28^. Typically algorithms are evaluated only on internal competition data and by participants themselves, which introduces a risk of overfitting and reduced likelihood of reproducibility. We addressed these limitations in our challenge design by using preregistration, blinded evaluation, full reproduction of algorithm results, independent validation of algorithms on external data and comparison with pathologists.

This study has limitations. First, for the validation phase we were limited to including 15 teams from the pool of 1,010. To ensure transparent selection of teams and minimizing potential bias in the external validation, we disclosed the selection criteria and process beforehand to all participating teams, included both score and algorithm descriptions as criteria, and performed the selection before running the analyses (Supplementary Methods 6).

Second, algorithm validation was restricted to the assessment of individual biopsies whereas, in clinical practice, pathologists examine multiple biopsies per patient. Future studies can focus on patient-level evaluation of tissue samples, taking multiple cores and sections into account for the final diagnosis. Third, this study focused on grading acinar adenocarcinoma of the prostate, and algorithm responses to other variants and subtypes of cancer, precancerous lesions or nonprostatic tissue were not specifically assessed. Although cases with potential pitfalls were not excluded, it is of interest to further examine algorithm performance on such cases (for example, benign mimickers, severe inflammation, high-grade prostatic intraepithelial neoplasia, partial atrophy) and to investigate which patterns consistently result in classification errors. Although not quantitatively assessed, an analysis of cases with frequent miscalls showed that these cases often contained patterns such as cutting artifacts, and different inflammatory and other biological processes—all common occurrences within pathology—that could have resulted in the algorithms’ miscalls. A comprehensive understanding of potential error modes is especially important when these algorithms leave controlled research settings and are used in clinical settings. Therefore, future research should more extensively assess what common tissue patterns in pathology routinely affect algorithm performance, whether they are the same patterns that are notoriously difficult for pathologists and how we can build safeguards to prevent such errors.

Fourth, algorithms were compared against reference standards set by various panels of pathologists. Although the gold standard in the field, relying on pathologists’ gradings introduces a risk of bias because algorithms could learn the grading habits of specific pathologists and not generalize well to other populations. To remedy this effect, panels of uropathologists established the reference standards of the EU internal and US external validation sets. Although these sets were graded in silo by different panels, we have shown that a majority-vote reference standard is highly consistent, even in a cross-continental setting (Supplementary Table 9). The EU external validation set was an exception, because a single uropathologist established the reference standard for that set. However, we observed high concordance between the grading by this pathologist and other pathologists when evaluated on the internal validation set (Supplementary Methods 2). For the training sets, we relied on reference standards extracted from clinical diagnostics, typically set by a single pathologist. Although unfeasible due to the high number of cases, a training reference standard based on multiple pathologists’ reviews could have potentially further increased algorithm performance.

Fifth, all the data were collected retrospectively across the institutions and the general pathologist reviews were conducted in a nonclinical setting, without additional clinical information available at the time of review. Sixth, despite the international nature of our evaluation (in terms of both pathologists’ practice and data sources), the countries involved were predominantly white, and demographic characteristics were not available for all datasets in the present study. Further investigation is required to validate the use of AI algorithms in more diverse settings^35,36^. Last, this study did not evaluate the algorithm grading’s association directly with radical prostatectomy or clinical outcomes.

We found that a group of AI Gleason grading algorithms developed during a global competition generalized well to intercontinental and multinational cohorts with pathologist-level performance. On all external validation sets, the algorithms achieved high agreement with uropathologists and high sensitivity for malignant biopsies. The performance exhibited by this group of algorithms adds evidence of the maturity of AI for this task and warrants evaluation of AI for prostate cancer diagnosis and grading in prospective clinical trials. We foresee a future where pathologists can be assisted by algorithms such as these in the form of a digital colleague. To stimulate further advancement of the field, the full development set of 10,616 biopsies has been made publicly available for noncommercial research use https://panda.grand-challenge.org/.

## Methods

### Study design

The study design of the PANDA challenge was preregistered^29^. We retrospectively obtained and de-identified digitized prostate biopsies with associated diagnosis from pathology reports from Radboud University Medical Center, Nijmegen, the Netherlands and Karolinska Institutet, Stockholm, Sweden (Extended Data Fig. 1 and Supplementary Methods 1, 2 and 3). At the start of the competition, participating teams gained access to this EU development set of 10,616 biopsies from 2,113 patients for training of the AI algorithms (Table 1 and Supplementary Methods 4). During the course of the competition, the teams could upload their algorithms to the Kaggle platform (Supplementary Methods 5) and receive performance estimates on a tuning set of 393 biopsies. Processing time was limited to 6 h and the maximum graphics processing unit (GPU) memory available was 16 GB.

By the competition closing date, each team picked two algorithms of their choice for their final submission, and the higher scoring of the two determined the team’s final ranking. The final evaluation was performed on an internal validation dataset of 545 biopsies, collected from the same sites as the development and tuning sets and fully blinded to the participating teams. Moreover, to obtain an independent internal validation set, all samples from a given patient were used for either development or validation.

After the competition on the Kaggle platform ended, all teams were invited to send in a proposal to join the validation phase of the study as members of the PANDA consortium. Joining the validation phase was fully voluntary and not a prerequisite for partaking in the competition. As a result, 15 teams were selected for further evaluation on two external validation datasets consisting of 741 and 330 biopsies, also fully blinded to the participating teams (Supplementary Methods 6 and 7). The first external validation set was obtained from two independent medical laboratories and a tertiary teaching hospital in the USA. The second external validation set was obtained from the Karolinska University Hospital, Stockholm, Sweden. All datasets consisted of both benign biopsies and biopsies with various ISUP GGs. For details on the inclusion and exclusion criteria, see Supplementary Methods 1 and Extended Data Fig. 1.

WSIs of the biopsies were obtained using four different scanner models from three vendors: 3DHISTECH, Hamamatsu Photonics and Leica Biosystems (Supplementary Table 1). The open source ASAP software (v.1.9: https://github.com/computationalpathologygroup/ASAP) was used to export the slides before uploading to the Kaggle platform.

The present study was approved by the institutional review board of Radboud University Medical Center (IRB 2016-2275), Stockholm regional ethics committee (permits 2012/572-31/1, 2012/438-31/3 and 2018/845-32) and Advarra (Columbia, MD; Pro00038251). Informed consent was provided by the participants in the Swedish dataset. For the other datasets, informed consent was waived due to the usage of de-identified prostate specimens in a retrospective setting.

### Reproducing algorithms and application to validation sets

All teams selected for the PANDA consortium were asked to provide all data and code necessary for reproducing the exact version of their algorithm that resulted in the final competition submission. For each algorithm, we collected the main Jupyter notebook or python script for running the inference, the specific Kaggle Docker^37^ image (https://github.com/Kaggle/docker-python) used by the team during the competition and any necessary associated files, including model weights and auxiliary code.

We replicated the computational setup of the competition platform and ran the algorithms on two different computational systems: Google Cloud and Puhti compute cluster (CSC—IT Center for Science, Espoo, Finland). On the Google Cloud platform, all algorithms were run using the original Docker images. On Puhti, the Docker images were automatically converted for use with Singularity^38^ (v.3.8.3). The algorithms and scripts provided by the teams were not modified except for minor adjustments required for successful run-time installation of dependencies on our computational systems. On Puhti, the algorithms had access to 8 central processing unit (CPU) cores, 32 GB of memory, 1 Tesla V100 32GB GPU (Nvidia) and 500 GB of SSD storage. On the Google Cloud platform, the algorithms had access to 8 CPU cores, 30 GB of memory, 1 Tesla V100 GPU 32GB and 10,000 GB of hard disk drive storage.

Before applying the algorithms on the external validation sets, we first validated that the Kaggle computational environment had been correctly replicated and the algorithms’ performance on our systems remained identical. To this end, we ran all algorithms on the tuning and internal validation sets on the two systems to reproduce the output generated during the competition on the Kaggle platform. By crosschecking the new results with the competition leaderboard, we additionally assured that the algorithms supplied by the teams were not altered after the competition or tuned to perform better on the external validation sets. The verification runs we performed on the Puhti cluster were used as the basis for all results reported on the internal validation set.

Some algorithms were nondeterministic, for example, because of test time augmentations with nonfrozen random seeds. We ran each of these algorithms five times and averaged the computed metrics.

After verification, we ran all algorithms on the external validation sets. For the US external validation set, we used the Google Cloud platform. For the EU external validation set, we used the Puhti cluster. This process was done independently of the teams and no prior information about the external datasets was supplied to the teams. The ISUP GG predictions of the algorithms on the cases were saved and used as input for the analysis.

### Statistical analysis

We defined the main metric as the agreement on ISUP GG with the reference standard of each particular validation set, measured using quadratic Cohen’s κ. To compare the performance of the algorithms with that of the general pathologists, we performed a two-sided permutation test per pathologist panel. The average agreement was calculated as the mean of the κ values across the algorithms and the pathologists, respectively. The test statistic was defined as the difference between the average algorithm agreement and the average pathologist agreement.

We calculated sensitivity and specificity on benign versus cancer-containing biopsies for all algorithms and individual general pathologists, based on the reference standard set by the uropathologists. To further understand how a representative pathologist and algorithm performed, we selected the pathologist and the algorithm with the median balanced accuracy (the average of sensitivity and specificity) as the representative pathologist and the representative algorithm, and reported the associated sensitivity and specificity. A representative pathologist or algorithm was used in favor of averaging across algorithms and pathologists for better estimates of performance. For the 95% CIs of the algorithms’ and pathologists’ performance metrics, we used bootstrapping across all algorithms or pathologists, with both the algorithm or pathologist and case as the resampling unit.

Analysis was performed using scripts^39^ written in Python (v.3.8) in combination with the following software packages: scipy (1.5.4), pandas (1.1.4), mlxtend (0.18.0), numpy (1.19.4), scikit-learn (0.23.2), matplotlib (3.3.2), jupyterlab (2.2.9) and notebook (6.1.5).

### Reporting Summary

Further information on research design is available in the Nature Research Reporting Summary linked to this article.

## Online content

Any methods, additional references, Nature Research reporting summaries, source data, extended data, supplementary information, acknowledgements, peer review information; details of author contributions and competing interests; and statements of data and code availability are available at 10.1038/s41591-021-01620-2.

## Supplementary information

Supplementary Information Supplementary methods, Tables 1–9 and Algorithm descriptions.

Reporting Summary

## Data Availability

The full development set, from here on named the PANDA challenge dataset, of 10,616 digitized de-identified hematoxylin and eosin-stained prostate biopsies, will be made publicly available for further research. The data can be used under a Creative Commons BY-SA-NC 4.0 license. To adhere to the ‘Attribution’ part of the license, we ask anyone who uses the data to cite the current article. The most up-to-date information regarding the dataset is available at the challenge website at https://panda.grand-challenge.org. Source data are provided with this paper.

## Source data

Source Data Fig. 2 Progression of algorithms’ performances (highest score and median) throughout the competition on the tuning and internal validation set.
Source Data Extended Data Fig. 7 Performance of individual algorithms compared with prior work on validation (sub)sets of those earlier works.

## Extended data

Extended data are available for this paper at 10.1038/s41591-021-01620-2.

## Code availability

Code that was used to generate the results of the various algorithms, and example code on how to load the images in the PANDA dataset is available at https://github.com/DIAGNijmegen/panda-challenge and 10.5281/zenodo.5592578. Algorithms were built using open source deep learning frameworks, including Pytorch (https://pytorch.org) and TensorFlow (https://www.tensorflow.org). The Docker image that all the algorithms were based on is available online at https://github.com/Kaggle/docker-python. Details on the availability of specific models and the code of the contributed algorithms can be found in the Supplementary algorithm descriptions.
