# Supplementary material for: Artificial intelligence for diagnosis and Gleason grading of prostate cancer: the PANDA challenge
Source: Nat Med. 2022 Jan 13;28(1):154–63. doi: 10.1038/s41591-021-01620-2 (PMC8799467; doi:10.1038/s41591-021-01620-2)
Supplement: Supplementary file 1 — Supplementary methods, Tables 1–9 and Algorithm descriptions. [file 41591_2021_1620_MOESM1_ESM.pdf]

nature portfolio

https://doi.org/10.1038/s41591-021-01620-2

Supplementary information

Artificial intelligence for diagnosis and Gleason grading of prostate cancer: the PANDA challenge

In the format provided by the authors and unedited

Supplementary Appendix

Supplementary appendix for “*Artificial Intelligence for Diagnosis and Gleason Grading of Prostate Cancer: the PANDA challenge.*”

Table of contents

Supplementary Methods **2**

Supplementary Tables **12**

Supplementary References **21**

Supplementary Methods

Section S1: Dataset inclusion and exclusion criteria

Development, tuning and internal validation set

For the development, tuning and internal validation set to be used in the PANDA challenge, we collected data from two different centers, namely Radboud University Medical Center (The Netherlands) and Karolinska Institutet (Sweden). These datasets were originally collected as part of two independent studies on automated Gleason grading.[1,2] For the purpose of this challenge, the datasets were merged and further refined. We will briefly reiterate the data collection here; further details can be found in the respective papers.

For the Radboud data, we retrieved all pathology reports dated between Jan 1, 2012, and Dec 31, 2017, for patients who underwent a prostate biopsy owing to a suspicion of prostate cancer (Extended Data Figure 1A).[1] Patients were randomly sampled based on the highest reported Gleason score mentioned in each report. Additionally, a set of reports was sampled which only mentioned benign biopsies. For each patient, a single hematoxylin and eosin-stained glass slide was selected for scanning. The selected glass slides were scanned using a 3DHistech Pannoramic Flash II 250 (3DHistech, Hungary) scanner at a pixel resolution 0.24 µm. Slides were then randomly sampled to be included in the development, tuning and internal validation sets. Randomization was stratified by patient and highest Gleason pattern present in the biopsy.

The data from Karolinska comes from the Stockholm-3 diagnostic trial that was conducted between May 28, 2012 and Dec 30, 2014 (Extended Data Figure 1B, ISRCTN84445406).[2–4] It was a prostate cancer screening-by-invitation trial of men aged 50–69 years living in Stockholm, Sweden. The purpose of the trial was to compare prostate specific antigen (PSA) to the Stockholm-3 model (S3M) for predicting the presence of cancer, and the criterion for referral to biopsy was either PSA above 3 ng/ml or a S3M probability of 10% or higher. A single pathologist (L.E.) assessed all biopsy cores in the trial and marked out the regions of cancer next to the tissue on the glass slide with a marker pen. A random sample from the biopsies included in the trial was taken, stratified on patient and the reported Gleason score to avoid including too many of the prevalent benign and low grade diseases. The selected slides were digitized at 20X magnification using two scanners: Hamamatsu C9600-12 (Hamamatsu Photonics, Hamamatsu, Japan) and Aperio ScanScope AT2 (Leica Biosystems, Wetzlar, Germany). The pixel size at full-resolution was 0.45202 µm (Hamamatsu) or 0.5032 µm (Aperio). Slides were then randomly sampled to be included in the development, tuning and internal validation sets. Randomization was stratified by patient and ISUP grade group.

To reduce the overall size of the various sets, and to achieve comparable resolution between centers, the Radboud images were downsampled and exported at a pixel spacing of 0.48 µm; the Karolinska images were exported at the original pixel spacing of 0.45 µm or 0.50 µm depending on the scanner. The images were exported as resolution pyramids with three levels representing downsampling factors of 1, 4 and 16 relative to the full resolution.

All images were converted to TIFF format with JPEG compression and a quality setting of 70.

US external validation set
The US external validation set consisted of retrospective cases from three different sources, and is described in detail in a prior study (Extended Data Figure 1C).[5] Briefly, cases were obtained from two medical laboratories and one tertiary teaching hospital. All tumor-containing cases available from the tertiary teaching hospital from 2005-2007 were included, and a fraction of the benign biopsies available were randomly sampled for inclusion. From the medical laboratories, all available ISUP grade group 4-5 cases were included in the study, and remaining benign and ISUP grade group 1-3 cases were randomly sampled for inclusion. One representative biopsy per case was included. Biopsies with non-gradable prostate cancer variants or quality issues preventing diagnosis were excluded from the dataset. Slides were digitized on an Aperio AT2 scanner (Leica Biosystems, Wetzlar, Germany) at a resolution of 0.25 µm/pixel ("40X magnification"). All images were converted to TIFF format with JPEG compression and a quality setting of 70.

EU external validation set
The EU external validation set comprised biopsy cores assessed by L.E. at the Karolinska University Hospital during 2018 (Extended Data Figure 1D). The set included all positive biopsy cores from all men diagnosed with an ISUP grade group 2, 3, 4, or 5 cancer as well as from a random selection of men diagnosed with ISUP grade group 1 cancer during that time period. In addition, the set included all cores from a random selection of men with only benign biopsies. This resulted in 330 slides from 73 men, scanned with a Hamamatsu NanoZoomer S360 C13220-01 (Hamamatsu Photonics, Hamamatsu, Japan). The pixel size at full resolution was 0.4604 µm. All images were converted to TIFF with JPEG compression and a quality setting of 70.

Section S2: Reference standard protocol

Development set - Radboud University Medical Center
For the cases in the development set from Radboud University Medical Center, the reference standard was determined based on the original pathology report. After scanning of the slides, trained non-experts assessed all slides and coarsely outlined each biopsy, assigning each with a Gleason score or the label 'negative' on the basis of the pathology report. If the pathologist report was inconclusive or lacked a description of individual biopsies, cases were flagged for a second review. If no match could be made in the second read, cases were excluded. To generate detailed label masks at gland-level, the biopsies were processed by a trained deep learning system that segmented the glands and assigned individual Gleason patterns to each gland.[1]

Development set - Karolinska Institutet
The cases from the Karolinska Institutet that were part of the development set were reviewed by a single uropathologist (L.E.). The review of the cases was performed on the original glass slides through a microscope. The uropathologist reported both the Gleason score and the ISUP grade group for each biopsy. Additionally, the uropathologist placed pen

marks on the glass slide alongside tumor tissue. Approximate label masks indicating benign and malignant tissue pixels were automatically generated based on the pen marks.[2]

Internal validation set and tuning set - Radboud University Medical Center
The reference standard for the Radboud University Medical Center cases that were part of the tuning and validation sets was determined in three rounds. In the first round, three uropathologists (C.H.v.d.K., R.V., H.v.B.) individually graded the cases digitally using the ISUP 2014 guidelines. For a number of cases, the majority vote was taken: cases with an agreement on ISUP grade group but a difference in Gleason pattern order, e.g., 5 + 4 versus 4 + 5; cases with an equal grade group but a disagreement on Gleason score; and cases for which two pathologists agreed while the third had a maximum deviation of one grade group. Cases with a disagreement on malignancy were always flagged for a second read in round two. In the second round, each biopsy without consensus was regraded by the uropathologist whose score differed from the other two. Additional to the pathologist's initial examination, the Gleason scores of the other pathologists were appended anonymously. Biopsies without consensus after round two were discussed in a consensus meeting.

Internal validation set and tuning set - Karolinska Institutet
The cases from the Karolinska Institutet that were part of the tuning set were reviewed by a single uropathologist (L.E.), similarly to the development set. The cases that were part of the internal validation set were initially reviewed by a single uropathologist (L.E.) on the original glass slides through a microscope. Cases initially indicated as benign were not re-reviewed. Cases indicated as malignant were divided between two other uropathologists (B.D. and H.S.), each reviewing 100 cases. In case of agreement between the first and the second review, the consensus ISUP grade group was assigned to the case. In case of disagreement, a third uropathologist (T.T.) reviewed the case. For cases that were indicated as malignant by all pathologists, the final ISUP grade group was assigned according to 2/3 consensus. If all three reviews were in disagreement, the case was excluded from the internal validation set. Any cases indicated as benign in the second or third review were excluded from the internal validation set. The second and third reviews were performed digitally using Cytomine, with all pathologists blinded to the other reviews.[6]

US external validation set
The US external validation set was reviewed by six uropathologists (M.B.A., A.J.E., T.K., M.Z., R.A., and P.A.H.) from 6 institutions with 18 to 34 years of clinical experience after residency (mean, 25 years). Reviews were first performed by 2 of the 6 uropathologists. A third uropathologist reviewed the specimens when there were discordances between the first two uropathologists. For cases without a majority opinion after 3 independent reviews, the median classification was used.

To limit the potential ambiguity of identifying Gleason patterns due to tissue processing procedure, such as tangential cuts of the specimen, two additional adjacent sections (levels) of the specimens were also available during review. Furthermore, one additional section per specimen was stained with the PIN-4 immunohistochemistry cocktail (P504S plus p63 plus high molecular weight cytokeratin) to assist the identification of cancer tissue. The three levels and the one PIN-4 stained slide were made available to the pathologists for establishing the reference standard. The biopsy at the middle level to be reviewed was highlighted.

EU external validation set
The cases from the Karolinska University Hospital that were part of the EU external validation set were reviewed by a single uropathologist (L.E.). The review of the cases was performed on the original glass slides through a microscope. The uropathologist reported both the Gleason score and the ISUP grade group for each biopsy.

To verify the consistency of the single-review reference standard of this set with the multi-review reference standard of the internal validation set, we compared L.E. to two other uropathologists who contributed to the multi-review reference standard for the samples from Karolinska Institutet. On the subsets of tumor-containing cases of the internal validation set graded by these two pathologists (B.D. and H.S.), we observed agreements (QWK) of 0.91 (n = 100) and 0.83 (n = 100) with L.E., respectively.

Reference standard consistency between EU and US uropathologist panels
The EU and US validation sets were independently reviewed by either US or EU-based panels of uropathologists. To assess consistency between the two reference standards we invited pathologists from the EU (C.H.v.d.K, H.v.B., R.V., L.E.) and US (M.B.A., A.J.E., T.K., R.A., P.A.H.) to review cases from the validation set they were not originally involved in assessing. The US pathologists reviewed 80 cases from the EU internal validation set, and the EU pathologists reviewed 83 cases from the US external validation set. Review of cases was performed using Cytomine[6].

After the independent reviews, we compared the individual uropathologists to the original reference standard. We additionally computed the pairwise agreement within the panels. Finally, we determined a majority vote based on the pathologist reviews and computed the agreement between this majority vote and the original reference standard. All agreements were quantified in QWK. If a majority vote achieves high concordance with the original reference standard, we can conclude that using multiple pathologists in a consensus/majority vote setting is a stable way of determining a reference standard.

The results of these cross-continental reviews are shown in Supplementary Table S9.

Section S3: Pathologist comparison review protocol

International pathologists comparison
As part of a previous study,[1] 100 biopsies were selected to be presented to a group of pathologists in an observer experiment. Benign cases were selected manually, controlling for a broad range of tissue patterns, including inflammation and (partial) atrophy. Cases containing cancer were sampled at random, stratified for ISUP grade group. Of these 100 cases, 70 were included in the internal validation set and used for the comparison to the panel of international pathologists.

The biopsies were made available through an online viewer, PMA.view (Pathomation, Berchem, Belgium), and distributed to an external cohort of pathologists. Cohort members were invited to participate in this study at the United States and Canadian Academy of Pathology 2019 annual meeting in Washington, DC, USA (March 16–21, 2019). Interested pathologists were subsequently asked to invite colleagues in their network who had

experience in Gleason grading. All pathologists who graded all biopsies were included. All cohort members had experience with Gleason grading, but to a varying degree. No time restriction was given, although we asked that they complete the grading within six weeks. In the original study, both pathologists and pathology residents were included. For the current study, only reads from pathologists were included. In total, the cohort consisted of 13 pathologists from eight countries (seven from Europe, six from outside of Europe).

US pathologists comparison
A subset of the US external validation set was reviewed by 20 US board-certified general pathologists. The pathologists reviewed the biopsies based on the 2014 ISUP grading guidelines.[7] Clinical information was not provided during grading and the pathologists were asked to review and grade biopsies as if they were reviewing a clinical slide in practice, without time constraints.

Section S4: Available training data during the competition

For algorithm development, teams could only use the competition dataset and publicly available datasets. For public datasets, usage was only allowed if teams disclosed this beforehand on the competition's public forum. By disclosing the use of external data, teams had no unfair advantage due to extra data availability. The use of private data, not available to other teams, was not allowed during the competition.

As part of the development set, we shared additional data besides the raw digitized biopsies to speed up development of the algorithms. As the reference standard, a comma-separated file (CSV) was supplied that mapped each biopsy ID to a Gleason score and ISUP grade group. Additionally, each training slide had an associated label mask that contained additional information about the tissue. The label masks were generated differently per institution and contained different types of labels.

For the slides originating from Radboud, each label mask outlined the tissue within the slide. Each pixel was labeled as either background, stroma/other tissue, benign epithelium, or one of the Gleason patterns 3, 4, or 5. The label masks were generated semi-automatically using a trained deep neural network and contained label noise. Additional details on how these masks were generated can be found in the respective paper.[1]

For the slides originating from Karolinska Institutet, the label masks were generated based on the annotations of the pathologist who graded the development set. Each label mask outlined the tissue areas within the slide. Additionally, for slides containing cancer, areas that contained malignant tissue were coarsely outlined in the mask. Further details on how these masks were generated can be found in the respective paper.[2]

Section S5: Kaggle competition platform

The PANDA challenge was hosted on Kaggle, one of the largest data science competition platforms. A competition typically runs for three months, during which participants or a team of participants can try to achieve the highest score in the competition's task. A Kaggle competition consists of two leaderboards: a public leaderboard visible during the competition

and a hidden private leaderboard. The public leaderboard gives teams an indication of how well their algorithm is performing during the competition. The blinded private leaderboard is used to determine the final competition ranking. Each algorithm submission is evaluated on both datasets, but only the score on the public leaderboard is shown to the teams. Because the private leaderboard is not shown, teams cannot directly tune their algorithm to score high on this leaderboard. For the PANDA challenge, the tuning set was used for the public leaderboard and the internal validation set for the private leaderboard. For the competition's final ranking, the teams could select two of the submissions entered during the competition, of which the highest scoring one was used for the ranking.

Competitions on Kaggle often have prize money for the top-performing teams to incentivize participants to sign up and reward them for the work done. For the PANDA challenge, the top three teams on the private leaderboard were awarded monetary prizes by Kaggle.

Entering the competition on Kaggle was free and open to everyone, after agreeing to the competition rules. After signup, participants had full access to the development set, which could be downloaded directly from the Kaggle website.

As part of the platform, every user had access to 30 free GPU hours per week for algorithm development. The development set was readily available when developing on the Kaggle platform, and no download was required. Additionally, participants were free to develop their algorithm offline on their hardware.

Participants were asked to submit a working version of their algorithm in the form of a Jupyter Notebook or a Python script to enter the competition. This notebook or script could have associated data that contained the learned parameters of the algorithm and any other required data sources. The submitted algorithm was required to be fully self-contained, which makes it possible to reproduce the results at a later stage. The algorithm had to be developed to process all cases supplied in a specific directory automatically. After submission, the platform populated this directory with the tuning set and internal validation set cases. This processing was fully blinded to the submitter of the algorithm. Processing time was limited to 6 hours when the algorithm used a GPU. Maximum GPU memory available was 16GB. To prevent cheating, algorithms did not have internet access during this evaluation, nor could they download or upload additional data. The number of allowed submissions was limited per team and participant to a maximum of three per day. The only information disclosed to the submitter was the public leaderboard score (and not the performance on individual cases).

Through a dedicated discussion forum, participants could discuss their algorithms and problems across teams. Participants often used the discussion forum to disseminate new ideas or share additional resources. Additionally, teams could share public versions of algorithms or code snippets for others to iterate on further. One of these public notebooks was created by the organizers to kick-start the competition and showcase the dataset.

Section S6: Methods to select the 15 teams for external validation

One month before the competition deadline, a post was placed on the competition's discussion forum to invite teams to join the PANDA consortium. Sign up was open to all

teams that submitted a working algorithm during the competition. The deadline for signing up was July 31st, 2020, eight days after the end of the competition. When signing up, teams were asked to report: team name, team members, the data requirements of their method, whether the algorithm was based on prior work or work of other teams, and a 1000 character abstract of their method. Additionally to the written submission, teams needed to give the challenge organizers access to their algorithm for review and further validation. In addition to the forum post, the organizers individually reached out to the top 30 teams of the competition's leaderboard and invited them to sign up.

After the deadline, five members of the organizer team (W.B., G.L., H.P., K.K., P.R.) individually reviewed all submissions and scored them on a five-point scale (1: strong reject, to 5: strong accept). The score was based on the overall method, originality, quality of the submission, and their algorithm's performance on the internal validation set. After scoring, all teams were discussed within the organizer team and a final ranking was established. Of all submissions, 15 teams (competition ranking in parentheses) were invited to join the PANDA consortium: *PND* (1), *Save The Prostate* (2), *NS Pathology* (4), *Kiminya* (5), *BarelyBears* (6), *ctrasd123* (7), *ChienYiChi* (8), *vanda* (10), *iafoss* (11), *Manuel Campos* (12), *Dmitry A. Grechka* (18), *KovaLOVE v2* (19), *Aksell* (20), *rähmä.ai* (27) and *UCLA Computational Diagnostics Lab* (28). Additionally, of these 15, eight teams were invited to present their method at the PANDA MICCAI workshop (October 8th, 2020, MICCAI 2020 virtual conference). All selected teams were included in the blinded validation on the external validation sets.

Section S7: Summary of participating teams' methods

All teams that were selected as part of the PANDA consortium were asked to report a summary of their method, including their training approach, dataset operations, and model architecture. The details on each team's methods are included in the supplementary algorithm descriptions. All algorithms selected for the independent validation made use of deep learning-based techniques. Some key design choices and algorithmic approaches adopted by the leading teams are discussed below.

End-to-end slide-level training

Many existing methods[1,2,5] employ so-called patch-based training, where a WSI is partitioned into smaller images, patches, that are used for model training. For each WSI, the predictions for these patches are then combined to obtain a slide-level prediction. This approach for training classifiers requires detailed annotations of the WSIs to obtain labels for individual patches or inferring patch-level labels from the slide-level label. In the PANDA dataset, coarse annotations were available for benign and malignant tissue, but ISUP grade groups were only provided per slide. As opposed to inferring patch-level labels from the slide-level ISUP grade group, end-to-end training emerged as the dominant strategy. This means that the entire WSI is treated as one data sample associated with a single target label. Due to their dimensions, processing full WSIs is, however, infeasible at high resolution due to limited GPU memory, and alternative methods were used to circumvent this.

A popular technique was proposed by the competition participant *iafoss,* and adopted and modified by several participants, including the winner of the competition (*PND*): In brief, this

approach consisted in selecting a subset of patches from a WSI based on simple filtering criteria, and processing these patches in a convolutional neural network (CNN) such that the feature representations of the patches are concatenated before they are being fed to the CNN's classification layers. This is a highly computationally efficient way for achieving end-to-end training of a single model producing a slide-level prediction and, to the best of our knowledge, a novel solution in the field.

As an alternative to selecting the representative tiles from a WSI based on relatively simple rules, some teams proposed the usage of additional CNN models to select the most informative tiles, typically operating on a low resolution version of the WSI (*Aksell, ChienYiChi, Save The Prostate, UCLA Computational Diagnostics Lab*). However, the top-ranking teams that also demonstrated the best generalization performance in external validation mainly relied on simple rules based e.g. on average pixel brightness for tile selection (*BarelyBears, Kiminya, Manuel Campos, NS Pathology, PND).*

Other training approaches included for example using CNNs as patch-wise feature extractors, followed by a recurrent neural network (*Dmitry A. Grechka*) or LGBM and XGBoost models (*UCLA Computational Diagnostics Lab)* for aggregating the patch-level feature representation to a WSI-level output. The success of the challenge participants in training models only based on slide-level labels is encouraging considering the sparsity of large datasets with patch- or pixel-level annotations. Slide-level labels are typically obtained as part of the clinical routine whereas collecting detailed annotations is usually a costly, additional process.

Data cleaning

Several teams used (semi-)automatic techniques to exclude low-quality samples from model training, removing for example patches or slides with penmarks (*Aksell, Dmitry A. Grechka, iafoss, rähmä.ai, Save The Prostate, UCLA Computational Diagnostics Lab*) and slides with inconsistencies between the slide-level labels and the provided label masks (*Save The Prostate, UCLA Computational Diagnostics Lab, vanda)*. Another data cleaning operation adopted by several teams (*Dmitry A. Grechka, Kiminya, UCLA Computational Diagnostics Lab, vanda)* was the detection of slides representing adjacent sections from the same sample via searching for similar images based on image hashing, and allocation of all such sections into the same fold during cross-validation to avoid information leakage between folds. However, many teams also reached top-ranking performance without reporting any data cleaning approaches.

Label denoising

A common denominator among most leading competition participants was the extensive use of label denoising. While label noise is present even in the validation sets due to human errors and the inherently subjective nature of Gleason grading despite consensus-based reference standards, the development set in particular contained substantial levels of label noise. For the cases of Radboud University Medical Center, the labels were generated semi-automatically. For the cases from Karolinska Institute, the reference standard was based on the assessment of a single pathologist, and the semi-automatically generated pixel-level labels indicated malignant regions only in an approximate manner. Many participants pointed out managing label noise during training as a key problem to solve during the competition.

For example, *BarelyBears* used Online Uncertainty Sample Mining and excluded 10% of training patches associated with the highest average loss values during training. *PND* and *Dmitry A. Grechka* identified samples with potentially erroneous labels after completing model training, based on the predicted ISUP grade group differing from the target label by more than a specified amount. Training was then repeated after exclusion of such samples. In the approaches of *iafoss* and *Save The Prostate*, the training labels were adjusted iteratively according to predictions by the trained model instead of excluding samples.

On the other hand, some teams achieved high performance on the internal validation set and/or top-ranking generalization to external data without reporting the use of any label denoising techniques *(Kiminya, Manuel Campos, NS Pathology, vanda)*. Despite the considerable improvement in performance on the internal validation set reported by some teams, label denoising can thus not be concluded to be useful for all types of models and training approaches.

Image resolution
While some teams experimented with multi-resolution approaches and used low resolution images for selecting regions of interest, virtually all of the proposed solutions used the intermediate resolution level of the input WSI for producing the final ISUP grade group predictions. This corresponds to approximately 2 μm pixel spacing (and a typical magnification of 5X), which can be considered a relatively coarse resolution. Three teams (*KovaLOVE v2, Save The Prostate, UCLA Computational Diagnostics Lab)* also utilized images on a resolution level obtained by downsampling the full resolution images by a factor of two from 0.5 μm to 1.0 μm pixel spacing, but these teams did not demonstrate any clear performance advantage to the teams that directly used the intermediate resolution level. Experimentation with full resolution images was even explicitly reported to not result in improved performance (*NS Pathology*). With a fixed memory budget, utilizing relatively low resolution patches allows increasing the physical area covered by each patch, the number of patches sampled per WSI and/or the batch size.

Data augmentation and normalization
A variety of data augmentation methods were adopted by the participants during model training in order to improve the robustness and generalization capability of the models. Simple spatial transformations such as random rotations and flips of the input patches were applied by all teams. Two of the top-performing teams (*BarelyBears, Save The Prostate)* applied augmentation additionally on slide-level by first applying a random affine transformation to the WSI before extracting patches.

In view of the variation in colors introduced by different scanners, data augmentation to make models invariant to small changes in color can be considered of special interest in the case of WSI data. Some form of color augmentation was applied by several teams (*Aksell, ctrasd123, iafoss, NS Pathology, PND, rähmä.ai, Save The Prostate, vanda),* including PANDA winner *PND,* and *NS Pathology*, who ranked 4th in internal validation but reached the best generalization performance on external data. Interestingly, however, another participant that ranked highly on both internal and external data (*Kiminya*) reported that they did not observe improved performance when using color augmentation, and hence did not include it in their final solution.

Test-time augmentation, that is, application of data augmentation while running predictions and averaging the results obtained for the perturbed versions of the same input, was also commonly applied (*Aksell, BarelyBears, Dmitry A. Grechka, iafoss, KovaLOVE v2, Manuel Campos, NS Pathology, vanda)*. In this case, the transformations consisted of standard operations such as rotations and flips of the images.

As opposed to augmentation, normalization was less commonly used. *BarelyBears* applied color normalization on patch level, and Global Contrast Normalization was used by *Dmitry A. Grechka* to standardize the contrast of patches.

CNN architectures
The participants mainly relied on neural network architectures representing EfficientNet variants (*BarelyBears, ChienYiChi, ctrasd123, KovaLOVE v2, Manuel Campos*, *NS Pathology, PND, Save The Prostate*) and ResNeXt variants (*Aksell, BarelyBears, iafoss, Kiminya, NS Pathology, rähmä.ai, Save The Prostate, UCLA Computational Diagnostics Lab, vanda*). Two participants also utilized the DenseNet121 architecture (*Dmitry A. Grechka, Manuel Campos*).

Ensembling
To improve performance and increase the algorithms' generalization ability, all of the 15 teams utilized ensembles of multiple models. Ensemble strategies differed from team to team, ranging from models trained using different hyperparameters, different patch selection strategies or different loss functions to a set of different neural network architectures combined into an ensemble. Overall, in many cases the ensembles represented diverse sets of design choices rather than training the same model with slightly different hyperparameters.

For example, *BarelyBears* used both the original data and refined data obtained after label denoising, and *PND* trained models with varying degrees of label denoising applied to the training data. Several teams combined classification models with regression models, as well as models trained with different loss functions. Overall, it was also typical to diversify the models in the ensemble by training them on different variations of the input data pre-processed in different ways, including different patch sizes.

Supplementary Tables

Table S1. Scanner details.

	EU Development set		EU Tuning Set		EU Internal Validation Set		US External Validation set	EU External Validation set
Source	Radboud University Medical Center Netherlands	Karolinska Institutet Sweden	Radboud University Medical Center Netherlands	Karolinska Institutet Sweden	Radboud University Medical Center Netherlands	Karolinska Institutet Sweden	Medical Laboratories, CA/UT, USA; Tertiary Teaching Hospital, CA, USA	Karolinska University Hospital Sweden
Scanning equipments	3DHistech Pannoramic Flash II 250 (3DHistech, Hungary)	Aperio AT2 (Leica, Germany) & Hamamatsu C9600-12 (Hamamatsu, Japan)	3DHistech Pannoramic Flash II 250 (3DHistech, Hungary)	Aperio AT2 (Leica, Germany) & Hamamatsu C9600-12 (Hamamatsu, Japan)	3DHistech Pannoramic Flash II 250 (3DHistech, Hungary)	Aperio AT2 (Leica, Germany) & Hamamatsu C9600-12 (Hamamatsu, Japan)	Aperio AT2 (Leica, Germany)	Hamamatsu C13220-01 (Hamamatsu, Japan)
Pixel spacing of original scanned slides	0.24 µm	0.50 µm and 0.45 µm	0.24 µm	0.50 µm and 0.45 µm	0.24 µm	0.50 µm and 0.45 µm	0.25 µm	0.46 µm
Pixel spacing of downsampled slides, available to the algorithms	0.48 µm	0.50 µm and 0.45 µm	0.48 µm	0.50 µm and 0.45 µm	0.48 µm	0.50 µm and 0.45 µm	0.50 µm	0.46 µm

Table S2. Individual algorithms' agreement with uropathologists on validation sets (quadratically weighted Cohen's kappa, 95% CI).

Team name	Internal validation set	US external validation set	EU external validation set	International pathologists comparison	US pathologists comparison
PND	0.941 (0.927-0.953)	0.882 (0.862-0.900)	0.890 (0.856-0.919)	0.905 (0.837-0.949)	0.856 (0.810-0.892)
Save the prostate	0.937 (0.924-0.948)	0.844 (0.820-0.866)	0.881 (0.849-0.908)	0.897 (0.835-0.940)	0.834 (0.781-0.873)
Kiminya	0.933 (0.911-0.950)	0.903 (0.886-0.919)	0.881 (0.844-0.913)	0.863 (0.719-0.947)	0.879 (0.836-0.911)
BarelyBears	0.933 (0.915-0.947)	0.872 (0.849-0.892)	0.890 (0.852-0.920)	0.847 (0.728-0.926)	0.845 (0.794-0.885)
Ctrasd123	0.932 (0.916-0.947)	0.853 (0.821-0.880)	0.879 (0.834-0.916)	0.882 (0.805-0.936)	0.805 (0.731-0.862)
ChienYiChi	0.932 (0.917-0.946)	0.851 (0.826-0.873)	0.886 (0.854-0.914)	0.875 (0.791-0.934)	0.809 (0.750-0.856)
NS Pathology	0.931 (0.911-0.947)	0.892 (0.869-0.911)	0.899 (0.868-0.924)	0.845 (0.727-0.927)	0.860 (0.811-0.897)
Manuel Campos	0.931 (0.915-0.944)	0.849 (0.819-0.874)	0.892 (0.854-0.923)	0.883 (0.806-0.937)	0.810 (0.738-0.864)
Vanda	0.930 (0.914-0.945)	0.888 (0.869-0.906)	0.880 (0.851-0.905)	0.894 (0.832-0.938)	0.860 (0.815-0.895)
Iafoss	0.930 (0.914-0.944)	0.861 (0.839-0.880)	0.824 (0.787-0.858)	0.856 (0.767-0.921)	0.831 (0.778-0.873)
UCLA CDx	0.929 (0.913-0.942)	0.860 (0.836-0.881)	0.848 (0.817-0.877)	0.868 (0.785-0.926)	0.814 (0.753-0.862)
KovaLOVE v2	0.928 (0.910-0.943)	0.814 (0.785-0.839)	0.880 (0.839-0.913)	0.882 (0.777-0.946)	0.774 (0.707-0.825)
Aksell	0.927 (0.910-0.942)	0.835 (0.800-0.863)	0.879 (0.851-0.904)	0.888 (0.827-0.931)	0.818 (0.761-0.863)
Rähmä.ai	0.926 (0.910-0.941)	0.869 (0.844-0.890)	0.865 (0.836-0.892)	0.866 (0.781-0.925)	0.832 (0.776-0.876)
Dmitry A. Grechka	0.926 (0.907-0.941)	0.851 (0.823-0.876)	0.740 (0.690-0.785)	0.897 (0.828-0.943)	0.794 (0.720-0.849)

Table S3. Tumor detection performance of individual algorithms on validation sets (sensitivity and specificity, 95% CI).

Team name	Internal validation set		US external validation set		EU external validation set		International pathologists comparison		US pathologists comparison	
	Sensitivity	Specificity	Sensitivity	Specificity	Sensitivity	Specificity	Sensitivity	Specificity	Sensitivity	Specificity
PND	99.1% (97.9-100.0)	93.7% (90.3-96.7)	99.0% (98.0-99.8)	75.2% (69.8-80.4)	96.4% (93.9-98.7)	90.7% (85.0-95.6)	98.2% (94.3-100.0)	100.0% (100.0-100.0)	98.5% (96.5-100.0)	67.5% (52.6-81.5)
Save the prostate	99.7% (99.0-100.0)	92.9% (89.2-96.1)	99.6% (99.0-100.0)	49.3% (43.3-55.3)	98.6% (97.0-100.0)	68.9% (60.0-77.7)	100.0% (100.0-100.0)	100.0% (100.0-100.0)	99.5% (98.4-100.0)	48.5% (33.0-63.5)
Kiminya	98.8% (97.4-99.7)	96.8% (94.3-99.0)	98.2% (96.8-99.2)	87.8% (83.7-91.7)	97.7% (95.6-99.5)	81.5% (74.0-88.6)	96.5% (91.1-100.0)	92.3% (75.0-100.0)	97.5% (95.0-99.5)	85.0% (73.0-95.1)
BarelyBears	99.4% (98.4-100.0)	96.4% (93.8-98.7)	99.2% (98.3-99.8)	70.1% (64.3-75.7)	97.3% (95.0-99.1)	89.8% (83.8-95.0)	98.2% (94.3-100.0)	100.0% (100.0-100.0)	99.0% (97.4-100.0)	72.5% (58.1-85.7)
Ctrasd123	98.5% (96.9-99.7)	98.2% (96.3-99.6)	97.5% (96.1-98.8)	83.5% (78.7-88.0)	94.1% (90.9-97.0)	95.4% (91.1-99.0)	98.2% (94.3-100.0)	100.0% (100.0-100.0)	97.5% (95.0-99.5)	85.0% (73.3-95.1)
ChienYiChi	99.4% (98.4-100.0)	92.8% (89.1-96.1)	98.6% (97.4-99.6)	70.5% (64.9-76.0)	97.7% (95.7-99.5)	84.3% (77.0-90.9)	100.0% (100.0-100.0)	92.3% (75.0-100.0)	98.5% (96.5-100.0)	70.0% (54.8-83.8)
NS Pathology	98.7% (97.4-99.7)	98.4% (96.6-99.7)	98.0% (96.7-99.1)	86.5% (82.4-90.5)	95.9% (93.2-98.1)	95.9% (92.0-99.1)	99.3% (97.8-100.0)	100.0% (100.0-100.0)	97.4% (94.9-99.4)	82.0% (70.0-92.4)
Manuel Campos	99.0% (97.8-99.9)	92.9% (89.4-96.2)	98.8% (97.8-99.6)	72.0% (66.7-77.4)	96.8% (94.4-99.1)	93.5% (88.5-97.8)	98.2% (94.3-100.0)	100.0% (100.0-100.0)	98.3% (96.5-99.6)	72.5% (57.9-85.7)
Vanda	99.7% (99.0-100.0)	92.8% (89.1-96.0)	99.4% (98.6-100.0)	80.7% (75.8-85.5)	99.5% (98.6-100.0)	68.5% (59.6-77.1)	100.0% (100.0-100.0)	92.3% (75.0-100.0)	99.0% (97.4-100.0)	80.0% (66.7-91.4)
Iafoss	98.8% (97.4-99.7)	91.9% (88.1-95.4)	99.8% (99.4-100.0)	60.6% (54.7-66.7)	99.1% (97.7-100.0)	74.1% (65.7-82.0)	98.2% (94.3-100.0)	100.0% (100.0-100.0)	99.5% (98.4-100.0)	57.5% (41.5-72.7)
UCLA CDx	98.8% (97.5-99.7)	93.7% (90.3-96.6)	98.6% (97.4-99.6)	75.2% (69.9-80.4)	100.0% (100.0-100.0)	65.7% (56.8-74.5)	100.0% (100.0-100.0)	92.3% (75.0-100.0)	97.0% (94.3-99.0)	75.0% (60.0-88.1)
KovaLOVE v2	99.7% (99.0-100.0)	93.7% (90.2-96.8)	98.4% (97.1-99.4)	52.0% (45.9-58.1)	96.8% (94.4-99.1)	89.8% (83.8-95.1)	100.0% (100.0-100.0)	100.0% (100.0-100.0)	98.0% (95.8-99.5)	45.0% (29.4-60.5)
Aksell	98.8% (97.4-99.7)	95.9% (93.2-98.3)	96.3% (94.6-97.9)	77.6% (72.5-82.6)	98.6% (96.9-100.0)	87.0% (80.2-93.0)	98.2% (94.5-100.0)	100.0% (100.0-100.0)	96.4% (93.6-99.0)	75.0% (60.5-87.5)
Rähmä.ai	98.1% (96.6-99.4)	92.8% (89.2-96.0)	99.0% (98.0-99.8)	74.0% (68.3-79.5)	99.1% (97.7-100.0)	60.2% (51.1-69.3)	100.0% (100.0-100.0)	92.3% (75.0-100.0)	98.0% (95.7-99.5)	72.5% (57.9-86.0)
Dmitry A. Grechka	98.7% (97.4-99.7)	92.6% (89.2-95.6)	96.9% (95.3-98.2)	82.0% (77.5-86.2)	100.0% (100.0-100.0)	44.1% (36.2-52.2)	100.0% (100.0-100.0)	92.3% (75.0-100.0)	97.2% (95.0-98.9)	83.5% (72.2-93.0)

Table S4. Ensemble algorithm performance. An ensemble was created by taking the majority vote for each case in the validation sets and computing the agreement of this ensemble with the reference standards.

Dataset / Metric	Internal validation set	US external validation set	EU external validation set	International pathologists comparison	US pathologists comparison
**Quadratically weighted Kappa (95% CI)**	0.940 (0.928-0.952)	0.892 (0.874-0.909)	0.899 (0.869-0.924)	0.887 (0.820-0.934)	0.870 (0.826-0.904)
**Sensitivity (95% CI)**	99.7% (99.0- 100.0)	99.2% (98.3-99.8)	98.2% (96.3-99.6)	100.0% (100.0-100.0)	99.0% (97.4-100.0)
**Specificity (95% CI)**	96.4% (93.8-98.7)	81.9% (77.1-86.6)	89.8% (83.8-95.1)	100.0% (100.0-100.0)	82.5% (69.7-93.5)

Table S5. Comparison of challenge algorithms to prior work. The performance of the teams' algorithms was computed on validation (sub)sets of earlier work.

	**EU Internal Validation Set (subset)**	**US External Validation set**	**EU External Validation set**
	Radboud University Medical Center Netherlands	Medical Laboratories, CA/UT, USA; Tertiary Teaching Hospital, CA, USA	Karolinska University Hospital Sweden
Number of cases	333	741	330
Average QWK among challenge teams (95% CI)	0.937 (0.919-0.952)	0.862 (0.840-0.884)	0.868 (0.835-0.900)
Prior works	0.926 (Bulten et al.)	0.863 (Nagpal et al.)	0.822 (Ström et al.)

Table S6. Pairwise agreements between pathologists who contributed to the reference standard.

	EU Internal Validation Set		US External Validation Set
Source	Radboud University Medical Center Netherlands	Karolinska Institutet Sweden	Medical Laboratories, CA/UT, USA; Tertiary Teaching Hospital, CA, USA
Pairwise mean agreement (QWK) all cases	0.926 (N=333)	N/A	0.907 (n=741)
Pairwise mean agreement (QWK) tumor cases only	0.853 (N=178)	0.876 (N=146)	0.809 (n=487)

Table S7. Clinical characteristics of the EU internal and external validation sets.

	**EU Internal Validation Set - Karolinska institutet, Sweden (N=82)**	**EU External Validation set - Karolinska University Hospital, Sweden (N=73)**
**Age, years**		
<54	3 (3.7%)	7 (9.5%)
55-59	9 (11.0%)	10 (13.7%)
60-64	19 (23.2%)	12 (16.4%)
65-69	48 (58.5%)	15 (20.5%)
>=70	3 (3.7%)	29 (39.7%)
**Prostate-specific antigen**		
<3 ng/mL	19 (23.2%)	-
3 - <5 ng/mL	37 (45.1%)	-
5 - <10 ng/mL	21 (25.6%)	-
>=10 ng/mL	5 (6.1%)	-

Table S8. Clinical characteristics from the two medical laboratories (ML1 and ML2) in the US external validation set. Clinical characteristics from the tertiary teaching hospital were not available.

	ML1	ML2
**Age at biopsy, years**		
<65	168 (44.2%)	129 (41.0%)
>=65	196 (51.6%)	181 (57.5%)
Not available	16 (4.2%)	5 (1.6%)
**PSA level at biopsy, ng/mL**		
<10	93 (24.5%)	198 (62.9%)
>=10	19 (5.0%)	68 (21.6%)
Not available	268 (70.,5%)	49 (15.6%)
**Reference standard grade group**		
No tumor	94 (24.7%)	147 (46.7%)
Grade group 1	147 (38.7%)	76 (24.1%)
Grade group 2	72 (18.9%)	44 (14.0%)
Grade group 3	46 (12.1%)	22 (7.0%)
Grade group 4	14 (3.7%)	6 (1.9%)
Grade group 5	7 (1.8%)	20 (6.3%)

Table S9. Additional crossover analyses by having experts reviewing datasets from another region. The algorithms were evaluated using both the original reference standard and the majority vote of the cross-continental experts.

	**Sampled EU Internal validation set**	**Sampled US external validation set**
	Radboud University Medical Center Netherlands, and Karolinska Institutet Sweden	Medical Laboratory, CA/UT, USA
Number of cases	80	83
Reference standard	For the Dutch part, consensus of 3 uropathologists.<br>For the Swedish part, 2-3 uropathologists per case.<br>(details in Supplementary Methods)	Majority vote of 3 US uropathologists (details in Supplementary Methods)
Additional participating pathologists from different region (cross-continental experts)	5 US pathologists	4 EU pathologists (3 from The Netherlands, 1 from Sweden)
Pairwise mean agreement (QWK) among cross-continental experts	0.852<br>(US pathologists)	0.887<br>(EU pathologists)
Agreement between individual cross-continental experts and reference standards (QWK)	0.880<br>(average of US pathologists vs EU reference standard)	0.911<br>(average of EU pathologists vs US reference standard)
Agreement between majority vote of cross-continental experts versus reference standards (QWK)	0.943<br>(majority vote US pathologists vs EU reference standard)	0.939<br>(majority vote EU pathologists vs US reference standard)

Supplementary References

1. Bulten, W. *et al.* Automated deep-learning system for Gleason grading of prostate cancer using biopsies: a diagnostic study. *Lancet Oncol.* **21**, 233–241 (2020).
2. Ström, P. *et al.* Artificial intelligence for diagnosis and grading of prostate cancer in biopsies: a population-based, diagnostic study. *Lancet Oncol.* **21**, 222–232 (2020).
3. Grönberg, H. *et al.* Prostate cancer screening in men aged 50-69 years (STHLM3): a prospective population-based diagnostic study. *Lancet Oncol.* **16**, 1667–1676 (2015).
4. Ström, P. *et al.* The Stockholm-3 Model for Prostate Cancer Detection: Algorithm Update, Biomarker Contribution, and Reflex Test Potential. *Eur. Urol.* **74**, 204–210 (2018).
5. Nagpal, K. *et al.* Development and Validation of a Deep Learning Algorithm for Gleason Grading of Prostate Cancer From Biopsy Specimens. *JAMA Oncol* (2020).
6. Marée, R. *et al.* Collaborative analysis of multi-gigapixel imaging data using Cytomine. *Bioinformatics* **32**, 1395–1401 (2016).
7. Gordetsky, J. & Epstein, J. Grading of prostatic adenocarcinoma: current state and prognostic implications. *Diagn. Pathol.* **11**, 25 (2016).

Supplementary Algorithm Descriptions

Supplementary appendix for *"Artificial Intelligence for Diagnosis and Gleason Grading of Prostate Cancer: the PANDA challenge."* This document contains algorithm descriptions of the selected teams that participated in the PANDA challenge. Each section describes a team's method, including their training approach, dataset operations, and model architecture. The descriptions were created by the respective teams.

Team: Aksell

Shujun He[1], Sejun Song[2], and Qing Sun[1]

[1]Texas A&M University
[2]Individual participant

Contact: shujun@tamu.edu, rvslight@gmail.com, sunqing@tamu.edu

Code and model availability: https://github.com/Shujun-He/PANDA

Abstract:
Here we describe a deep learning approach for automatic prostate cancer (PCa) diagnosis that incorporates tile segmentation on low-resolution images, self-attention, and multi-task learning. Our methods are conceptually simple but effective, leading to accurate diagnosis of PCa despite noisy and imbalanced training data. In addition, our model is interpretable due to usage of self-attention and will only improve as more multi-labeled data accumulates.

Data preparation:
Whole slide images (WSI) were padded and divided into square tiles of identical dimensions, with three color channels (see Fig. A1). Tiles with pen marks and low prostate cell count were removed in the process. We sorted the tiles based on two criteria to get two sets of tiles for each WSI. First, since white tiles and tiles with large white spaces tend to have high pixel intensity, we sorted by the average pixel intensity and retained (usually 36) tiles with the lowest values. This method should be credited to the competition participant *Iafoss* and we refer to these tiles as intensity tiles. Second, we used the provided masks indicating cancer cells to train a tile-level segmentation model on the lowest resolution level images to make binary predictions (containing cancer or not) for each tile. Using the tile segmentation model, we selected tiles that were most likely to contain cancer, referred to as segmentation tiles.

Training setup:
Following tile selection, we proceeded to train our models using the tiles obtained from medium resolution WSIs. Our final submission included two classification models and three regression models, each of which included a convolutional neural network (CNN) backbone (ResNeXt-50 [1] pre-trained on ImageNet), pooling, and a classification/regression output layer, and some of which had a self-attention function before the final pooling and output layer. For models with self-attention, generalized mean pooling was used to pool each tile into a feature vector, and following self-attention, max mean concat pooling was used to pool all feature vectors into one before the linear layer that produced a regression/classification output. Dropout of 0.5 was used before the last layer in all models.

We used the Adam [2] optimizer with an initial learning rate of 1.0×10^{-4} with 0.1 decay at 36 and 42 epochs for a total of 45 epochs or a one cycle schedule for 30 epochs. For the 45-epoch training schedules, we used downsampled half resolution tiles (usually 36x128x128 or

20x112x112 for segmentation tiles) for the first 10 epochs for faster training and then full resolution tiles (usually 36x256x256 or 20x224x224 for segmentation tiles) for the final 35 epochs. Two of the models were trained on intensity tiles and three on segmentation tiles. Gradient accumulation was used to accommodate small batch sizes due to GPU memory constraint and the effective batch size was always kept between 32 and 64. We started with a cross validation (CV) strategy with iterative stratification to ensure class balance between folds. However, later we discovered that there was little to no difference on the public test set between single fold models and averaging five fold models, so for faster experiments, we used only one fold models for validation.

For data augmentation, we used a combination of cutout [3], random rotation, random color change, random transposition, and random flipping. We found this combination to be an effective way to prevent overfitting. More aggressive augmentation such as cutmix [4] did not result in any improvement.

Model parameters:
Total parameters ResNeXt50 with self-attention: 28.2 million (all trainable).
Total parameters ResNeXt50 without self-attention: 25.5 million (all trainable).

Inference setup:
During inference, first we processed WSIs into tiles and selected tiles based on pixel intensity or probability of containing cancer. Then we applied 8x test time augmentation (vertical/horizontal flip, and transpose). The classification models' outputs were taken as scalar predictions and averaged together with predictions from regression models. Although some of our models were trained with multiple tasks (ISUP grade group, majority Gleason pattern, minority Gleason pattern), only the outputs for the ISUP grade group were used in making predictions.

Acknowledgements:
We would like to thank *Iafoss* for graciously sharing his method of tile generation and selections, which proved to be simple and effective.

References:
1. Xie, S., Girshick, R., Dollar, P., Tu, Z., & He, K. (2017). Aggregated Residual Transformations for Deep Neural Networks. 2017 IEEE Conference on Computer Vision and Pattern Recognition (CVPR). doi:10.1109/cvpr.2017.634
2. Diederik P. Kingma, & Jimmy Lei Ba. (2015) Adam, a Method for Stochastic Optimization, ICLR 2015
3. Terrance DeVries, & Graham W. Taylor, Improved Regularization of Convolutional Neural Networks with Cutout, arXiv:1708.04552, 2017
4. Sangdoo Yun, Dongyoon Han, Seong Joon Oh, Sanghyuk Chun, Junsuk Choe, Youngjoon Yoo, CutMix: Regularization Strategy to Train Strong Classifiers with Localizable Features, arXiv:1905.04899, 2019

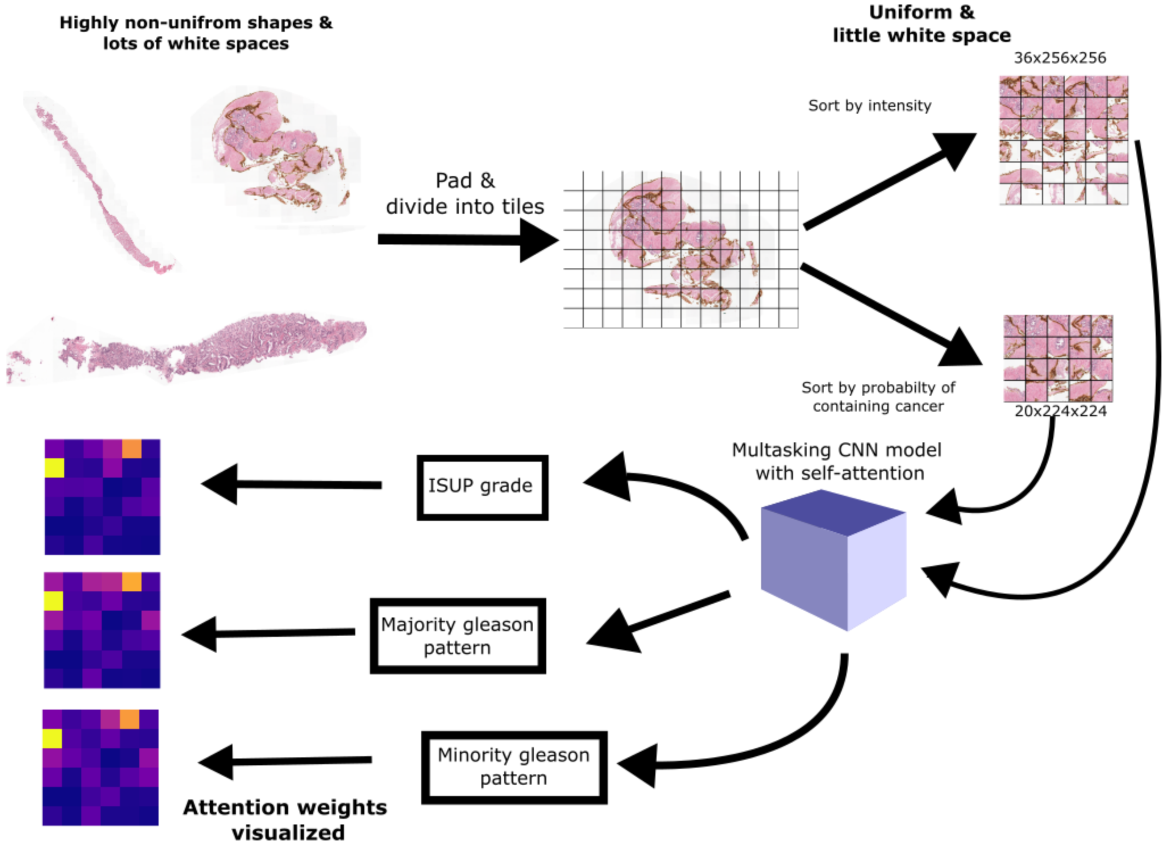

Figure A1: Overview of the solution of *Aksell*. WSIs were first processed into tiles based on two different criteria. Then a multitasking CNN model was trained and used to make predictions on ISUP grade group, majority Gleason pattern, and minority Gleason pattern. The model also provides interpretability through the use of self-attention, which can highlight regions of interest relevant for the model's prediction.

Team: BarelyBears

Hiroshi Yoshihara[1, 2], Taiki Yamaguchi[3], Kosaku Ono[4], Tao Shen[5]

[1]Department of Health Informatics, Kyoto University, Kyoto, 6068303, Japan
[2]Aillis Inc., Tokyo, 1000005, Japan
[3]Preferred Networks Inc., Tokyo, 1000004, Japan
[4]Nowcast Inc., Tokyo, 1020073, Japan
[5]School of Biological Science and Medical Engineering, Southeast University, Nanjing, 210000, People's Republic of China

Contact: hiroshi.yoshihara@aillis.jp

Code and model availability: https://github.com/analokmaus/kaggle-panda-challenge-public

Abstract:
We developed an automated Gleason grading system, which is an ensemble model of four multi-instance learning (MIL) networks. A MIL network consists of a feature extractor which extracts features from patches obtained from a WSI, and a head which concatenates and pools all the features and predicts the ISUP grade group. Various backbones were used in the feature extractors. Networks were trained with Online Uncertainty Sample Mining (OUSM) [1], or with Mixup [2] in order to improve robustness to label noise. The ensemble model trained noise-robustly showed better performance compared to the model trained in an ordinary manner.

Data preparation:
We extracted input patches to the networks from the intermediate level WSIs without any preprocessing. Patches were selected based on low pixel intensity. Due to the presence of noisy labels in the training dataset, ensemble denoising was performed for the original training dataset. We trained networks with different random seeds, and identified samples which are likely to have noisy labels, in other words, samples associated with a high average loss. We excluded the top ten percent of samples with highest average loss from each class. Some of the networks were trained with the denoised dataset. No external data were used.

Training setup:
Our grading system consists of four MIL networks. All MIL networks have a common structure: a patch feature extractor which extracts patch-wise features, and a head which concatenates and pools all the features extracted from all patches and predicts the ISUP grade group. SE-ResNeXt50, SE-ResNeXt101 [3], and EfficientNet-b0 [4] pretrained on the ImageNet dataset were used in the feature extractors. Consistent rank logits framework [5] was used in the heads because of the rank-consistent nature of the ISUP grade group.

All patches were extracted from intermediate resolution level WSIs. Data augmentation was applied to both WSIs and patches. First, affine transforms were applied to the WSIs before

patches were extracted. Then, affine transforms, horizontal and vertical flip, random dropout, and colour normalization were applied to the patches. Input shape, in other words the number and size of patches, is shown in Table A1.

In order to train networks robustly to label noise, OUSM [1] was used. OUSM assumes that noisy samples have higher loss, and thus removing *k* samples with high loss from each mini-batch should improve the network's robustness. With a batch size of 12, *k* was set to 1 or 2. Mixup [2], which is a well known data augmentation algorithm, also improved robustness to noisy labels.

All networks were trained with the Adam [6] optimizer, a learning rate of 0.0002, and a batch size of 12. A 5-fold CV was conducted. The learning rate was halved after every 3 consecutive epochs without improvement on the validation set, and we stopped training after 15 epochs without improvement. Network configurations are shown in Table A1.

Table A1: Network configurations used by *BarelyBears*.

Network #	Input shape	Feature extractor	Dataset	Denoising
1	64*224*224	SE-ResNeXt50	denoised	OUSM k=1
2	64*224*224	SE-ResNeXt50	original	OUSM k=2
3	64*224*224	SE-ResNeXt101	denoised	OUSM k=1
4	36*256*256	EfficientNet-b0	original	Mixup alpha=0.4

Model parameters:
Parameters (trainable parameters)
Network #1: 27611125 (27611125)
Network #2: 27611125 (27611125)
Network #3: 49006645 (49006645)
Network #4: 6602345 (6602345)
Total: 110831240 (110831240)

Inference setup:
All networks in Table A1 were applied to the preprocessed patches extracted from a WSI. The result from each network is a five dimensional vector whose *i*-th element refers to the probability of the prediction being higher than ISUP grade group *i*, and in total four such vectors are obtained. A temporary prediction was calculated by averaging the sum of each vector. Note that this temporary prediction is not discrete but a continuous value. In order to improve the robustness of the predictions, four inputs with random data augmentations applied as described in the training setup were passed to the model, and the temporary predictions for them were averaged. The predicted ISUP grade group was obtained by discretizing the temporary

prediction, based on the best set of thresholds maximizing quadratic weighted kappa, precalculated during training.

Acknowledgements:
The authors are grateful to Aillis Inc. for providing computational resources.

References:

1. Xue C, Dou Q, Shi X, Chen H, Heng P. Robust Learning at Noisy Labeled Medical Images: Applied to Skin Lesion Classification. In: 2019 IEEE 16th International Symposium on Biomedical Imaging (ISBI 2019). ; 2019:1280-1283. doi:10.1109/ISBI.2019.8759203
2. Zhang H, Cisse M, Dauphin YN, Lopez-Paz D. mixup: Beyond Empirical Risk Minimization. In: International Conference on Learning Representations. ; 2018.
3. Hu J, Shen L, Sun G. Squeeze-and-Excitation Networks. In: Proceedings of the IEEE Conference on Computer Vision and Pattern Recognition (CVPR). ; 2018.
4. Tan M, Le Q V. EfficientNet: Rethinking Model Scaling for Convolutional Neural Networks. In: Chaudhuri K, Salakhutdinov R, eds. Proceedings of the 36th International Conference on Machine Learning, {ICML} 2019, 9-15 June 2019, Long Beach, California, {USA}. Vol 97. Proceedings of Machine Learning Research. PMLR; 2019:6105-6114.
5. Cao W, Mirjalili V, Raschka S. Rank-consistent Ordinal Regression for Neural Networks. 2019:1-8. http://arxiv.org/abs/1901.07884.
6. Diederik P. Kingma, & Jimmy Lei Ba. (2015) Adam, a Method for Stochastic Optimization, ICLR 2015

Team: ChienYiChi Team

Jianyi Ji[1], Arnaud Roussel[1], Kairong Zhou[1]

[1]Individual participant.

Contact: jijianyi1993@gmail.com, arnaudrousselqc@gmail.com

Code and model availability:
https://github.com/ChienYiChi/kaggle-panda-challenge
https://github.com/arroqc/pandacancer_kaggle

Abstract:
WSIs of biopsies have billions of pixels. The common way to deal with this is to divide the image into a grid of tiles and select relevant ones for the task. To efficiently select tiles from the WSI, we trained a model with an attention layer [1] over these tile candidates. The top-ranked *k* of the tiles were processed by another CNN model. Finally, all features were aggregated with a NetVLAD [2] layer before the final output.

Data preparation:
We used the intermediate resolution level images from the TIFF files. We first divided the image into a grid, and saved tiles that are not empty (based on the sum of non-white pixels) in PNG format, to be used for training and inference.

Training setup:
The models were trained in two steps. First, a model with an attention layer was built [1]. The attention layer was added after a CNN backbone which extracts features from each tile. This attention layer learns attention weights to compute a weighted average feature vector for the bag of tiles. Then, classification is performed based on that vector. We used 128 tiles (with the least amount of white pixels) for model input and a tile size of 256x256x3 pixels. Once trained, we reused that model up to the attention layer to compute the weights of all the tiles we have. We selected the top 16 tiles according to the attention weight. Then, we trained new models to make the final prediction which uses these 16 tiles as input.

The backbones used were all EfficientNet b0 and b4 [3]. For the second step models, some used bags of tiles as input, while others used tiles stacked into squares. To aggregate the independent tile features generated by the backbone model into a single vector, we applied Average/Maximum Pooling layer or NetVLAD [2] layer before the final output layer.

The Adam optimizer [4] with a cosine annealing learning rate scheduler was used for model fitting. The following image augmentations were used: flipping, shifting. The network was developed using PyTorch.

Model parameters:

1. first model: 4,834,434 (trainable 4,834,434)
2. second model: 56,343 (trainable 56,343)

Inference setup:

Each model used in the ensemble had a similar setup as for training. The input images were extracted from the TIFF file, then cropped into non-overlapping tiles with dimensions of 256x256x3 pixels. We then sorted them by the sum of the pixel value of these tiles in descending order and selected the top 128. We passed these tiles through the first model (EfficientNet with attention layer) and got their attention weights. We selected the 16 highest weighted tiles and fed them to the second models.

The second model generates tile features, aggregates them and outputs the final result. There are two types of predictions in our solution depending on the model: regression and ordinal regression. For regression output, we used a list of intervals [0.5, 1.5, 2.5, 3.5, 4.5] to split the output into different groups. For ordinal regression, the output was passed through a sigmoid layer and we simply summed up outputs (5 output nodes) to get the final group value. We then aggregated all the different predictions from our ensemble and averaged the predictions followed by rounding to the nearest integer.

References:

1. Ilse M, Tomczak J, Welling M. Attention-based Deep Multiple Instance Learning https://arxiv.org/pdf/1802.04712.pdf
2. Relja Arandjelović, Petr Gronat, Akihiko Torii, Tomas Pajdla, Josef Sivic. NetVLAD: CNN architecture for weakly supervised place recognition https://arxiv.org/abs/1511.07247.pdf
3. Mingxing Tan, Quoc V. Le. EfficientNet: Rethinking Model Scaling for Convolutional Neural Networks https://arxiv.org/abs/1905.11946.pdf
4. Diederik P. Kingma, & Jimmy Lei Ba. (2015) Adam, a Method for Stochastic Optimization, ICLR 2015

Team: ctrasd123

Tianrui Chai[1], Nina Weng[2]

[1]School of Computer Science and Engineering, Beihang University, Beijing, China
[2]DTU Compute, Technical University of Denmark, 2800 Kgs. Lyngby, Denmark

Contact: trchai@buaa.edu.cn

Code and model availability:
Training code: https://github.com/ctrasd/Panda-2020-gold-medal-solution
Testing code: https://www.kaggle.com/ctrasd123/panda-singlenet-submit
Models: https://www.kaggle.com/ctrasd123/pandamodels

Abstract:
We developed an automated Gleason grading algorithm based on an ensemble of efficient-net [1] models. To make most of the data, we split the data into 5 folds, preprocessed the data with different methods and trained efficient-nets separately on each set. In order to adapt the input to the networks, we proposed two approaches of tiling to deal with the large size of the images. Finally, when running predictions, we averaged the results of eight networks to get the final prediction score.

Data preparation:
We used the middle resolution level of the TIFF images and applied two approaches of tiling to prepare our data: 1) We tiled our images into 256 x 256 pixel slices and sorted them according to the sum of pixel values. Finally, we selected 36 slices containing the largest area of prostate tissue to form the final image of size 1536 x 1536; 2) We processed the images with a hierarchy of dimensions of 256 x 256, 192 x 192 and 128 x 128. First, we cut 24 slices with a size of 256 x 256 and then cut 16 slices of 192 x 192 size in the remaining area. Finally, we cut 12 slices of 128 x 128 size and combined the three sizes of slices to form the final 1536*1536 image.

Training setup:
Our deep learning system consists of 8 efficient-nets trained on the 1536 x 1536 images which are generated as described above. We split the training data into 5 folds and trained different nets with different folds. Experimentation showed that there is no difference in the performance of efficientnet-b0, b1, b2 and b7. Thus, we simply used efficientnet b0 as our backbone and GeM pooling [2] as the final pooling layer. Six of the eight nets were trained on the images generated by the first tiling approach and the other two nets were trained on the images generated by the second tiling approach. The Adam optimizer [3] was used with a learning rate of 0.0003, and a batch size of 8 on four 2080Ti GPUs (2 per GPU). We adopted the CosineAnnealingLR method to adjust the learning rate for one round. We transformed the six category task into six binary category tasks and used binary cross entropy as the loss.
Our neural networks were developed using PyTorch. Before inputting the images into the networks, we made some random adjustments in brightness, brightness, saturation, and hue on

the training dataset. We also used a random horizontal flip and random vertical flip to augment our data.

Model parameters: 32,121,880 (trainable 32,121,880).

Inference setup:
For each image, our single neural network outputs 6 predicted probabilities to indicate whether the image reaches the corresponding ISUP grade group. When a probability is higher than 0, the image is considered to have reached the corresponding grade group. We average the predicted probabilities of our eight nets to get the final predicted probability, and get the final predicted label. Only the highest ISUP grade group is taken as the final class. For example, [1,1,0,0,0,0] means reaching ISUP grade group 1 and [1,1,1,0,1,0] means reaching ISUP grade group 4.

References:

1. Tan, Mingxing, and Quoc Le. "EfficientNet: Rethinking Model Scaling for Convolutional Neural Networks." International Conference on Machine Learning. 2019.
2. Radenović, Filip, Giorgos Tolias, and Ondřej Chum. "Fine-tuning CNN image retrieval with no human annotation." IEEE transactions on pattern analysis and machine intelligence 41.7 (2018): 1655-1668.
3. Diederik P. Kingma, & Jimmy Lei Ba. (2015) Adam, a Method for Stochastic Optimization, ICLR 2015

Team: Dmitry A. Grechka

Dmitry Grechka[1]

[1] Individual participant.

Contact: dmitry@grechka.family

Code and model availability:
Training code: https://github.com/dgrechka/PANDA-Challenge
Trained model: https://zenodo.org/record/4053040

Abstract:
The automated prostate biopsy grading system consists of a CNN and a recurrent neural network (RNN) chained together to predict the ISUP grade group of a tissue sample. The WSIs are split into a grid of smaller square tiles. Each tile containing tissue is first mapped into a feature vector by applying the CNN (DenseNet121 [1]). Then, the feature vectors (presented as a sequence) are passed to the RNN (GRU [2] units) to evaluate the presence of cancerous tissue and to assign a corresponding ISUP grade group.

Data preparation:
The intermediate resolution level WSIs' (pixel size approx. 2 µm) pixel values were inverted, that is, each value was replaced by 255 (white) minus the original pixel value, and the images were split into a grid of 256 x 256 pixel tiles. Tiles at the image boundaries were zero padded to the size of 256 x 256. Tiles without tissue or with a lot of pen marks (identified by maximum pixel brightness less than 10, or mean green to red channel ratio less than 1.2) were discarded.

Next, Global Contrast Normalization (GCN) [3] was applied. Contrast was evaluated as the standard deviation of pixel intensity across tiles originating from a single image. The normalized tiles were saved in RGB24 pixel format representing a brightness range of two standard deviations from the mean brightness. Finally, the tiles were downsampled to 224 x 224 size with Gaussian resampling.

The dataset was cleaned from images that were considered as wrongly labeled or too hard to learn. There were three iterations of dataset cleaning. Each iteration included network training, evaluation of prediction error, removal of samples with high prediction error (absolute difference between predicted and ground truth ISUP grade group greater than or equal to 2.5). On each iteration, every sample of the original dataset was (re)evaluated for candidacy for removal.

To prevent occasional removal of valid samples, there was an independent model training and evaluation for two CV folds. Only the samples exhibiting high prediction error across both CV folds were removed. Dataset cleaning resulted in 271 removed samples.

The training dataset was augmented by rotating the initial images (before tile splitting) by angles multiple of 72°. Resulting tiles were saved in the TFRecords file format to provide fast training data ingestion by the TensorFlow framework.

Training setup:
The model input was a sequence of tiles originating from a single WSI. The tiles were sorted by descending mean pixel intensity (tiles with extensive blank area go last). The same number of tiles were extracted from all WSIs (by cycling or truncating the tile sequence). Each tile was randomly augmented by vertical/horizontal flip, 90-degree rotation, then transformed by DenseNet121 to obtain a feature tensor with shape 7x7x1024. This was followed by Max2D pooling to get the feature vector of 1024 elements. Two densely connected layers with 256 and 128 units were applied sequentially to reduce the number of feature vector dimensions. After that, the sequence of feature vectors was passed to two GRU layers with 96 and 64 units respectively. The 64 elements output of the later GRU layer was passed to the dense layer with scalar output. This layer with sigmoid activation scaled by 5.0 acted as a ISUP grade group regression head.

The backbone (DenseNet121) was initialized with weights pretrained on ImageNet, later layers were randomly initialized. The RMSprop optimizer was used to minimize LogCosh loss for regression.

Training images that were serial sections of the same tissue block were clustered together with the help of the imagehash library. Image clusters underwent 5-fold CV splits, also keeping the Gleason score frequencies in the training sets the same as in the validation sets.

For each CV fold the training consisted of three stages. Each subsequent stage started with the previous stage's results. The common settings of all the stages were: batch size 2, starting learning rate 1.0×10^{-4}, gradient clipping by norm 1.0, dropout rate 0.4, L2 regularization coefficient 1.0×10^{-4}, learning rate reduce factor 0.1. The stages differed in the following: backbone weights frozen/unfrozen, tile sequence length, early stopping patience, learning rate reduction patience and the monitored metrics. For the specific values, see the *train_phase_config.json* files of the computational experiments *"37c"* and *"40c"* in the published source code.

Model parameters:
7,429,057 parameters (7,345,409 trainable).

Inference setup:
The system determines the ISUP grade group for a biopsy as the mean of six predictions generated by an ensemble: three models with identical network architecture and different learned parameters applied for an image with and without initial image rotation (see Table A2). As the network outputs the ISUP grade group as a continuous value in the range between 0.0 and 5.0, the average value of the predictions is finally rounded to the nearest integer. The input tiles are extracted, filtered and processed the same way as described in the "Data Preparation" section.

Table A2: Prediction ensemble configurations used by *Dmitry A. Grechka*.

Model parameters set		initial image rotation angle
Training experiment name	CV fold number	
37c	2	0.0°
37c	2	22.5°
37c	3	0.0°
37c	3	45.0°
40c	5	0.0°
40c	5	67.5°

The experiments 37c and 40c of model training differed in the number of initial dataset cleaning iterations (see “Data Preparation” section): 37c had two iterations resulting in the removal of 245 images, while 40c had one more iteration (three iterations in total), resulting in the removal of 271 images. To map the CV fold number to image clusters used as a validation set, see the *data/trValSplits/5foldClusteredGleasonScore/* directory of the published source code.

Acknowledgements:
I want to thank the developer team of Alpheus - computational experiments management tool (https://github.com/itislab/alpheus), for implementing my feature requests during this and other machine learning competitions.

References:

1. Huang G, Liu Z, Van Der Maaten L, Weinberger K. Densely Connected Convolutional Networks. 2017 IEEE Conference on Computer Vision and Pattern Recognition (CVPR). 2017. doi:10.1109/cvpr.2017.243
2. Cho K, van Merrienboer B, Gulcehre C et al. Learning Phrase Representations using RNN Encoder–Decoder for Statistical Machine Translation. Proceedings of the 2014 Conference on Empirical Methods in Natural Language Processing (EMNLP). 2014. doi:10.3115/v1/d14-1179
3. Goodfellow I, Bengio Y, Courville A. Deep Learning.; 2016. 442-444

Team: Iafoss

Maxim V. Shugaev[1]

[1]Department of Materials Science and Engineering, University of Virginia, 395 McCormik Road, Charlottesville, Virginia 22904-4745, USA

Contact: mvs9t@virginia.edu

Code and model availability:
Training code examples: https://github.com/iafoss/PANDA
Trained model: https://www.kaggle.com/iafoss/panda-init-class-model1

Abstract: We developed an end-to-end system relying on a novel Concatenate Tile pooling method, which allowed training based on labels assigned to entire WSIs while using a tile-based approach. The computational cost of training was reduced by more than 10 times in comparison to training on full images. The training efficiency was further improved by the use of a newly proposed tile cutout method. Progressive label self-distillation and removal of noisy labels was applied to handle the substantial level of label noise in the training data.

Data preparation: The intermediate resolution level of the WSIs was padded and divided into 128 x 128 pixel tiles. The tiles produced from each WSI were sorted based on the sum of all pixels, and 128 tiles with the lowest sum were selected and saved as PNG images (128 x 128 x 128 tile setup). Since white color corresponds to background, this procedure selects 128 tiles with the largest tissue area. In addition, 12 x 128 x 128 tiles cut from the lowest resolution level were used for pretraining the models.

One of the main components of the challenge was dealing with noisy labels. Specifically, the labels of the Radboud training dataset were generated semi-automatically, and the concordance of the semi-automated method with experts was estimated by the authors to be 0.853 in terms of quadratically weighted kappa [1]. Therefore, the training procedure included two stages. At the first stage a progressive label self-distillation was run. First, a set of models was trained on the original data, and out of fold predictions $dr_1$and dk_1 were used to produce adjusted labels as $lr_1 = (2lr + dr_1)/3$ and $lk_1 = (3lk + dk_1)/4$ for the Radboud and Karolinska data, respectively, where lr and lk are the original labels. Next, the procedure was repeated producing adjusted labels as $lr_2 = (4lr + dr_1 + dr_2)/6$ and $lk_2 = (6lk + dk_1 + dk_2)/8$. The weights were selected to have approximately 1200 and 200 adjusted labels differing from the original ones for Radboud and Karolinska data, respectively. At the second stage, images with $|lk_2 - lk| > 0.5$, $|lr_2 - lr| > 0.5$, and $|dr_2 - lr| > 0.75$and images having pen marks were dropped, and training was performed on a smaller dataset containing 8700 images with reliable labels.

Training setup: The deep learning system is illustrated in Fig. A2. Specifically, instead of passing an entire image as an input, N tiles are selected from each image based on the number of tissue pixels and passed independently through the convolutional part. The outputs of the convolutional part for each tile are concatenated into a single large feature map followed by pooling and a fully connected head. Since any spatial information is eliminated by the pooling layer, the Concatenate Tile pooling approach is nearly identical to passing an entire image through the convolutional part, excluding predictions for nearly empty regions, which do not contribute to the final prediction, and shuffling the remaining outputs into a square map of smaller size.

Elimination of empty regions reduces the computational cost of training and inference as well as GPU memory requirements. In addition, use of tiles having the same size provides an effective way to build batches out of images having different sizes and aspect ratios. Finally, since the prediction is generated based on a set of tiles corresponding to an image, rather than independent individual tiles, labels assigned to the images can be used directly during training (end-to-end manner). There is thus no need to perform an intermediate step with prediction of Gleason pattern masks for individual tiles followed by evaluation of the WSI's ISUP grade group as a postprocessing step.

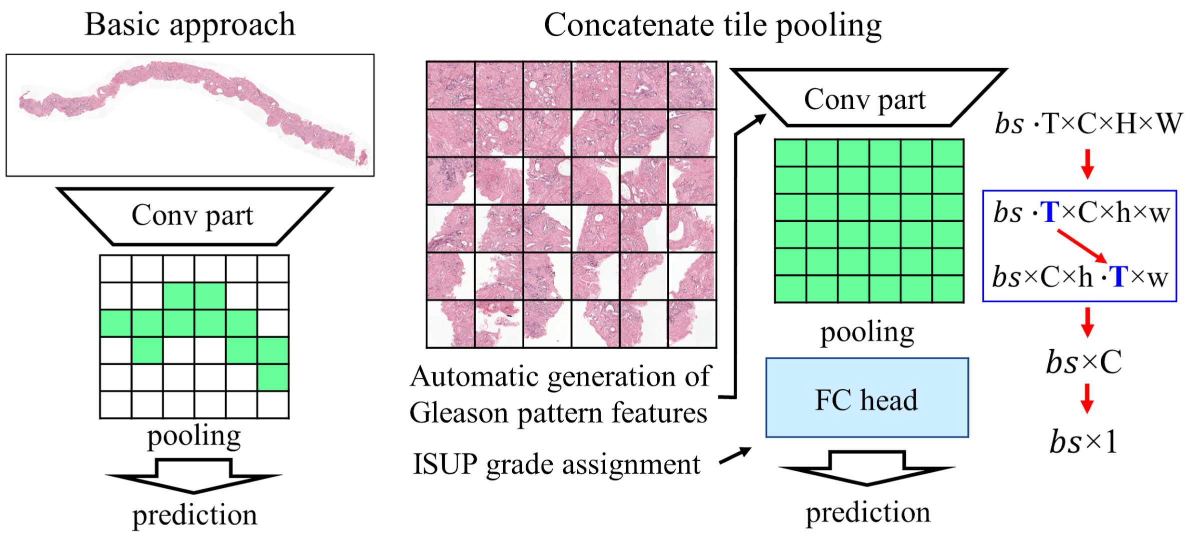

Figure A2: Overview of the solution of *iafoss*.

The final model was based on a ResNeXt50 backbone pretrained on 1 billion images in a semisupervised manner [2]. Concatenation of Average and MaxPooling from the final feature map produces features based both on the relative areas of the Gleason patterns present and the most aggressive Gleason pattern, which are required to assign the ISUP grade group. The fully connected head, meanwhile, learns the procedure of assigning ISUP grade groups based on the features built by the convolutional part. In addition to ISUP grade group, the network produces an auxiliary output for Gleason score used to provide additional guidance to the network during training.

Kappa loss based on an expression proposed in [3] was minimized during training. In addition, CrossEntropy loss was applied to the Gleason score auxiliary output with the weight of 0.08. The Kappa loss requires a sufficiently large batch size for convergence. Therefore, progressive resizing was utilized: each model was first trained on 12 x 128 x 128 tiles produced from the lowest resolution level with a batch size of 64, and then training was continued on 128 x 128 x 128 tiles produced from the intermediate resolution level with a batch size of 8.

The augmentation used during training included transformations based on dihedral symmetry group, shift, rotation, and rescaling as well as random changes of brightness, contrast, hue, saturation, and value. In addition, a novel method, tile cutout, was utilized. The idea of this augmentation is similar to a regular cutout, i.e. replacement of a part of the input image with a black square, but it allows substantially reducing the computational cost and GPU memory requirements during training if utilized with Tile Concatenate pooling. Specifically, for training on a 128 x 128 x 128 tile setup only 96 tiles are randomly selected at each time, which effectively corresponds to a random removal of 25% of the input image. This removal also reduces the training time and GPU memory usage by 25%, in contrast to a regular cutout not affecting the computational cost of training. Finally, an augmentation based on tile selection was utilized. By adding an extra padding of 64 to the original images on horizontal, vertical, and both directions, each original image produced 4 sets of different tiles, which were saved and randomly selected during training.

Model parameters: 25,082,183 for each model.

Inference setup: During inference, 128 x 128 x 128 tiles were extracted from the intermediate resolution level for each WSI and passed as an input to the models. Due to the time limit imposed on inference, only 6 out of 8 dihedral symmetry group operations were utilized for test time augmentation: original image, horizontal flip, vertical flip, diagonal flip, rotation by 90 degrees, and rotation by 90 degrees + horizontal flip. The final prediction was produced by the majority voting ensemble of 8 models trained with 4 fold train/validation split.

References:

1. Bulten W, Pinckaers H, van Boven H, et al. Automated deep-learning system for Gleason grading of prostate cancer using biopsies: a diagnostic study. Lancet Oncol 2020;21(2):233–41.
2. I. Zeki Yalniz, Hervé Jégou, Kan Chen, Manohar Paluri, Dhruv Mahajan: Billion-scale semi-supervised learning for image classification. May 2019. arXiv:1905.00546
3. David Vaughn, Derek Justice: On The Direct Maximization of Quadratic Weighted Kappa. September 2015. arXiv:1509.07107

Team: Kiminya

Raphael Kiminya[1]

[1]Individual participant, Nairobi, Kenya.

Contact: kiminyaraphael@gmail.com

Code and model availability: Code and model not shared.

Abstract:
The developed CNN-based system relies on a core network based on the ResNeXt50 [1] backbone, with pre-trained weights from semi-supervised and semi-weakly supervised ImageNet models [2]. The network was trained with a tile-based approach, where the most informative regions of each slide were extracted and individually passed through the CNN. The outputs of the individual tiles were then concatenated and used to predict the ISUP grade group of the whole image.

Data preparation:
There were a number of possible duplicates and serial sections from the same tissue block. To avoid information leakage during training, it was important to identify and handle these duplicates. From the lowest resolution level of the WSIs, JPEG images were extracted, and *Imagehash* was used to calculate the hash representing each image. Image pairs with a similarity score greater than 0.9 were considered duplicates. A mapping between each image and its duplicates was used to split the data during training such that all duplicates were in the same fold. This minimized leakage during training by ensuring that duplicates of each sample were either in the training or validation set.

Due to the large size and sparsity of tissue pixels on the WSIs, training on the whole images was not ideal. The optimized tiling approach, where the most informative tissue patches were extracted from each image based on the color density of the pixels, was thus adopted. Two datasets were generated, with tile sizes of 256 and 384 from the resolution level 1 of each WSI.

Training setup:
The solution was based on the concatenate tile pooling idea, where N tiles were selected from each image and passed independently through a CNN's convolutional layers. The outputs were then concatenated and the classification layer applied on the whole image.

There was a performance trade-off between selecting more tiles or a larger batch size - selecting more tiles would have speeded up the learning but meant selecting a lower batch size, which made the training unstable. A random sample of n tiles from the top N tiles was used for each epoch.

Each model was trained with the backbone frozen for one epoch, then unfrozen and fine tuned on all layers for 15 epochs with learning rate of *3e-04* and weight decay of *1e-04*. Label binning with BCE loss function was used. The kappa score was tracked after each epoch and the best performing model saved. The training parameters used for the different models are summarized in Table A3.

Color and lighting based data augmentation did not improve the model performance, and augmentation was thus limited to affine and distortion transforms - rotations, flips, zoom, warp - all based on the default fastai transforms.

Table A3: Summary of the various combinations of tile options and training parameters used by *Kiminya*.

Model	Tile size	Model input size	N	K	Batch size	CV folds
resnext50_32x4d_ssl	256	192 x 192	28	40	10	6
resnext50_32x4d_ssl	256	256 x 256	32	40	6	10
resnext50_32x4d_swsl	384	384 x 384	14	24	6	5

Model parameters:
Total parameters: 25,081,157 x 21 models.

Inference setup:
Similar to training, inference was performed on the medium resolution level of the WSIs. Tiles were extracted for each image and *N=28* tiles selected from the top *K=40* most informative tiles. The ISUP grade group was then predicted for each image and the predictions averaged over the models.

Acknowledgements:
Find duplicates: https://www.kaggle.com/appian/panda-imagehash-to-detect-duplicate-images
Optimized tiling: https://www.kaggle.com/akensert/panda-optimized-tiling-tf-data-dataset
Concat tile pooling: https://www.kaggle.com/iafoss/panda-concat-tile-pooling-starter-0-79-lb
Label binning BCE: https://www.kaggle.com/haqishen/train-efficientnet-b0-w-36-tiles-256-lb0-87

References:
1. Xie, S., Girshick, R., Dollar, P., Tu, Z., & He, K. (2017). Aggregated Residual Transformations for Deep Neural Networks. 2017 IEEE Conference on Computer Vision and Pattern Recognition (CVPR). doi:10.1109/cvpr.2017.634
2. I. Zeki Yalniz, et al. “Billion-scale semi-supervised learning for image classification”. May 2019.
   arXiv:1905.00546 [cs.CV]

Team: KovaLOVE v2

Vassili Kovalev[1], Dmitry Voynov[1], Valery Malyshev[1], Elizabeth Lapo[1]

[1]The United Institute of Informatics Problems of National Academy of Sciences of Belarus, Minsk, 220012, Belarus

Contact: malyshevalery@gmail.com

Code and model availability:
Training and inference code (inference code to be run on Kaggle):
https://github.com/Fever07/PANDA-challenge-code
Pre-trained EfficientNet-B0 and manually trained models: https://zenodo.org/record/4017792

Abstract:
We developed a neural network-based ISUP grade group predictor. We trained the network using groups of tiles extracted from the WSIs as input. Finally, we made use of several inference techniques including shifted tile slicing, building an ensemble of models, and test-time augmentation. As a result, every input WSI acquired 32 possibly different scores and the final prediction was made taking all of them into account.

Data preparation:
Every WSI in the dataset was saved at a pixel spacing of 0.96 µm. Images in this resolution were cut into non-overlapping tiles of 256 x 256 pixels. From these tiles, we selected the ones that contained the largest areas of prostate tissue. Then we composed a so-called canvas of tiles by placing 16, 25, or 36 selected tiles into a square collage and stored it as a single image. As a result, for each original image, we retrieved three canvases with dimensions of 1024/1280/1536 pixels, respectively. This procedure made it possible to replace the large WSIs with canvases that were small enough to be used as input to the neural network.

Training setup:
We used the pre-trained EfficientNet-B0 [1] as a backbone for our neural network models. The Gleason grading problem was posed as an ordinal regression problem. Thus, we configured the networks to predict 5 output values ranging from 0 to 1 and trained them using binary cross-entropy loss. Since the input canvases were relatively large, we trained the networks with relatively small batch sizes in the range from 2 to 4 depending on canvas dimensions. We used the Adam optimizer [2] with a starting learning rate of 0.0003, a warm-up factor of 10 with a single warm-up epoch, and a cosine annealing scheduler. Experiments showed that 30 epochs were sufficient for stable and effective neural network training. We validated the training process on a single fold due to its high computational complexity. To increase the quality and robustness of the network both tile-level and canvas-level image augmentation techniques were used. They included transposition as well as vertical and horizontal flipping.

Model parameters: 4,013,953 (trainable 4,013,953).

Inference setup:
At first, the scanned tissue of the biopsy was cut into tiles, as described above. Second, the tiles were placed into a single canvas, which represents the diversity of tissue types. Then, we fed the canvas to the trained neural network. Finally, the output vector was summed and rounded to retrieve the prediction for the ISUP grade group of the WSI.

We applied the following additional techniques during inference:
1. *Shifted tile slicing.* In parallel with the original slicing procedure, we carried out slicing by shifting the starting point by 64/128/192 pixels diagonally towards the bottom-right corner. This process resulted in four different groups of tiles, forming four different canvases. The predictions performed for the four canvases were averaged to obtain the ISUP grade group.
2. *Models ensembling.* We chose four trained neural networks to build an efficient ensemble. The mean value of the predictions of the models was taken as the ISUP grade group.
3. *Test-time augmentation.* In addition to the original image we also predicted the ISUP grade group for its augmented version. To this end, we used a 90 degree rotation to produce the augmented image and averaged the two predictions.

Applying these techniques, we obtained a total of 32 ISUP grade group predictions for each input WSI, and averaged them to obtain the final prediction.

Acknowledgements:
The authors are deeply grateful to D. Pavlenko for his help in finding and analyzing additional materials that were necessary to complete this work.

References:
1. Tan, Mingxing, and Quoc Le. "EfficientNet: Rethinking Model Scaling for Convolutional Neural Networks." International Conference on Machine Learning. 2019.
2. Diederik P. Kingma, & Jimmy Lei Ba. (2015) Adam, a Method for Stochastic Optimization, ICLR 2015

Team: Manuel Campos

Manuel Campos[1]

[1] Individual participant, Madrid, Comunidad de Madrid, Spain

Email address: coreacasa@telefonica.net

Code and model availability:
Training code: https://www.kaggle.com/coreacasa/code-base-training-one
https://www.kaggle.com/coreacasa/code-base-training-two-enets
https://www.kaggle.com/coreacasa/code-base-training-two-densenet
Inference code: https://www.kaggle.com/coreacasa/12th-place-solution-quick-save-inference
Dataset Weights: https://www.kaggle.com/coreacasa/pandaenetb042x256x256x3

Abstract:
The algorithm is based on the EfficientNet [1] and DenseNet [2] network families. Each WSI was decomposed into small tiles and then recomposed into a new input that includes all the tiles in a more reliable representation. To handle label noise: 1) we trained our models on the full dataset without validation but with a completion based on a timid exhaustion of the loss function, and 2) we performed model ensembling.

Data preparation:
We applied the Concatenate Tile Pooling method [3], where instead of passing an entire WSI as an input, N tiles are selected from each image based on the number of tissue pixels and passed independently through the convolutional part of the models. Basically, for each WSI and its corresponding mask, we extracted smaller resolution tiles and composed a mosaic of N such tiles as the input. We chose a tile size of 256 x 256 pixels with 3 channels and N of 36, 42 or 48 tiles, producing inputs of 36 x 256 x 256 x 3, 42 x 256 x 256 x 3 and 48 x 256 x 256 x 3 [4].

Training setup:
(I) We experimented with CV (see *code-base-training-one*), training on one fold for 60 epochs to monitor the loss value evolution, and using 40 epochs for the other folds. The evolution and final values of the loss function (MSE) were rather similar across different folds. Ensembling the models did not lead to improvement on the public leaderboard score, and the models' performance was uneven when introduced into an external ensemble. This is caused by label noise and the sensitivity of the QWK metric to even small variations in MSE when evaluated on the test data.

We applied the following training parameters: Size Image 256, Size Tiles 256, Tiles 42, Augmentation horizontal and vertical flips with p=0.5, Validation StratifiedKFold 5 on ISUP grade groups, Arch EfficientNetB0, Convolutional Base's Weight Imagenet trainable, On Top GlobalAveragePooling2D, Dropout(0.5), Dense(1024), Output Dense(1) regression objective

Loss mean_squared_error, Optimizer Adam, Leaning Rate 5e-04 init, Reduce LR decreasing 0.5 with patience 3 epochs, Save weights only with best validation loss epochs, Batch Size 64.

(II) We did not attempt removing noisy labels from training data. In general, we are not in favor of losing any existing information, although in principle it could be harmful by elevating the non-regular part of a data generating process. While preferable to removing data, we also did not attempt to transform the data. Instead, we opted for training the models with the full dataset in order to prevent the possible existence of more noise in some folds than in others, which probably would have increased the variability in the inference results.

We trained 3 members of the EfficientNet family (see *code-base-training-two-enets*) and applied the following changes to the parameters of the approach (I) presented above: Tiles 48, Validation Art Validation on instinct, Arch EfficientNetB0, EfficientNetB1 and EfficientNetB2, Convolutional Base's Weight Noisy Student trainable, Output Dense(5,activation='sigmoid) ordinal regression objective, Loss sigmoid_cross_entropy_with_logits, Leaning Rate custom with 5up, 3sustain, 0.8decay, Limits LR 1e-05min, 4e-04max, Save weights only with best loss epochs, Batch Size 32, Epochs 60. Finally, we trained 1 member of the DenseNet family (see *code-base-training-two-densenet*). Additional changes to the training parameters described above were: Arch Densenet121, Convolutional Base's Weight Imagenet trainable, Epochs 40.

Model parameters:
Not registered, see technical information of [1] and [2]. The models were always trained without freezing the convolutional base weights pre-trained on Imagenet, with more layers added on top.

Inference setup:
Our inference procedure was based on the diversity of Architectures, Tiles and Test Time Augmentations. From the training processes above, the following pairs of models and inputs were available: EfficientNetB0-42x256x256x3, EfficientNetB0-48x256x256x3, EfficientNetB1-48x256x256x3, EfficientNetB2-48x256x256x3 and DenseNet121-48x256x256x3. Further, from the public notebooks in the competition we picked EfficientNetB0-36x256x256x3 [5] and EfficientNetB1-36x256x256x3 [6].

Moreover, we applied the following Test Time Augmentations: --Type A: 5xTTA deterministic,1xoriginal, 1xTranspose, 1xVerticalFlip, 1xHorizontalFlip, 1xTranspose->VerticalFlip->HorizontalFlip, --Type B: 4xTTA pseudo deterministic 1xoriginal, 1xVerticalFlip, 2xHorizontalFlip(p=0.5)->VerticalFlip(p=0.5) and --Type C: 2xTTA random 2xHorizontalFlip(p=0.5)->VerticalFlip(p=0). (II) White Padding Tile Extraction, -- x add zero pad and 1x add 256 pad, that is, 2 different extractions for ALL the images. (III) Model Selection and Final Ensemble, --see quick-save-inference-solution for details.

Acknowledgements:
We reserve this special section to highlight the work of those competitors who have made our final solution better, 1) because their ability was not present in my initial knowledge or 2)

because their performance improves together with the experience of mine. I mean, in no order of priority, [Salman], [Qishen Ha], [RAHUL SINGH INDA], [Iafoss]. Please, check the links in References.

References:

1. Gao Huang, Zhuang Liu, Laurens van der Maaten, Kilian Q. Weinberger: Densely Connected Convolutional Networks, https://arxiv.org/abs/1608.06993v5
2. Mingxing Tan, Quoc V. Le: EfficientNet: Rethinking Model Scaling for Convolutional Neural Networks, https://arxiv.org/abs/1905.11946v5
3. Iafoss, https://www.kaggle.com/iafoss
4. Salman, https://www.kaggle.com/micheomaano
5. Qishen Ha, https://www.kaggle.com/haqishen
6. RAHUL SINGH INDA, https://www.kaggle.com/rsinda

Team: NS Pathology

Noriaki Ota[1], Shinsuke Yamaoka[1]

[1]Systems Research & Development Center, Technology Bureau, NS Solutions Corp., Kanagawa, 220-8401, Japan

Contact: ota.noriaki.4qp@jp.nssol.nipponsteel.com, yamaoka.shinsuke.5ke@jp.nssol.nipponsteel.com

Code and model availability:
Training code:
https://github.com/hirune924/lightning-hydra
https://github.com/sinpcw/kaggle-panda-public
Trained model:
https://www.kaggle.com/sinpcw/panda-model-4
https://www.kaggle.com/hirune924/pandamodel2

Abstract:
We used tiling as preprocessing. The size of the tiles was determined such that the tissue area in the image would fit evenly into 16 tiles. We also applied random coefficients to the size of the tiles during training, which acts as data augmentation and prevents the model from overfitting to the training data. We also used SyncBN to stabilize the behavior of batch normalization during training. We used hard voting instead of averaging as an ensemble method.

Data preparation:
We extracted the inputs for the model from the middle resolution level of the WSIs. Experimenting on the highest resolution level did not lead to any improvement. We divided the image into a grid of 16 tiles and combined them to form a single image, which was then resized to 2048 x 2048 (16 x 512 pixels x 512 pixels). Tile size was determined according to the percentage of tissue area in the image, as shown in the equation below, so that the tissue fitted evenly into the 16 tiles. We used 2.0 for the scaling factor, except during training.

$$\sqrt{height \times width \times (1.0 - ratio\ of\ white\ area) \times scaling\ factor \div 16} \qquad (1)$$

Training setup:
We used the following models: EfficientNet b5 [1] x 4, Se-ResNeXt 101-64x4d [2] x 2, Se-ResNeXt 101-32x4d x 2, ResNeSt 101e [3] x 2, GeM[4] + EfficientNet b3 x 1. Data augmentation was used to increase the robustness of the network. We randomly changed the scaling factor in equation (1) from 0.5 to 3.5 as data augmentation. In addition, the following augmentation procedures were used: RandomGridShuffle, GridDropout, Cutout, GridDistortion, Flip, RandomHueSaturationValue, and RandomBrightnessContrast. Batch size of 8 was used and Synchronized Multi-GPU Batch Normalization was used to stabilize the behavior of Batch Normalization. MSE Loss was used as the loss function and Adam [5] was used as the

optimizer. CyclicLR was used to schedule the Learning Rate, and the Learning Rate was varied from 5e-6 to 1e-4 during one epoch. We stopped training when there was no longer any improvement in the public leaderboard score.

Model parameters:
EfficientNet b5: 28,342,833 (trainable 28,342,833).
Se-ResNeXt 101-64x4d: 86,186,033 (trainable 86,186,033).
Se-ResNeXt 101-32x4d: 46,908,465 (trainable 46,908,465).
ResNeSt 101e: 46,228,065 (trainable 46,228,065).
GeM + EfficientNet b3: 10,697,770 (trainable 10,697,770).

Inference setup:
Our method determines the ISUP grade group by aggregating the inference results of the above models. We also use RandomGridShuffle and Flip (horizontal and vertical) for test time augmentation to obtain three predictions per model. For each inference result, we assign a ISUP grade group by applying threshold values of 0.5, 1.5, 2.5, 3.5 and 4.5. The class getting the highest number of votes in each of those majority votes is the final inference result. If the maximum number of votes does not exceed 1/3 of the total number of votes, we calculate the average of all the model inferences and assign the ISUP grade group based on the thresholds above. The rationale for combining the two methods is that a majority vote alone reduces performance when the inference results are disparate, while using only the average has a negative effect when some inference results are far off.

References:

1. Tan M, Le QV. EfficientNet: Rethinking model scaling for convolutional Neural Networks. arXiv [csLG]. 2019. http://arxiv.org/abs/1905.11946.
2. Hu J, Shen L, Albanie S, Sun G, Wu E. Squeeze-and-Excitation Networks. IEEE Trans Pattern Anal Mach Intell. 2020;42(8):2011-2023.
3. Zhang H, Wu C, Zhang Z, et al. ResNeSt: Split-Attention Networks. arXiv [csCV]. 2020. http://arxiv.org/abs/2004.08955.
4. Gu Y, Li C, Xie J. Attention-aware generalized mean pooling for image retrieval. arXiv [csCV]. 2018. http://arxiv.org/abs/1811.00202.
5. Diederik P. Kingma, & Jimmy Lei Ba. (2015) Adam, a Method for Stochastic Optimization, ICLR 2015

Team: PND

Yusuke Fujimoto[1], Kentaro Yoshioka[2]

[1]Rist Inc., Tokyo, 153-0063, Japan
[2]Wireless System Lab., Toshiba Corp., Kawasaki, 211-0014, Japan

Contact: yukkyo12221222@gmail.com, kyoshioka47@gmail.com

Code and model availability: https://github.com/kentaroy47/Kaggle-PANDA-1st-place-solution

Abstract:
We developed an automated Gleason grading algorithm with simple label noise reduction. All neural networks were based on EfficientNet-b0 and b1 [1], trained with 5 CV folds. Our solution was based on three steps: first, we train a network with the original training labels. The main challenge of this competition was the presence of considerable label noise. To deal with that problem, we chose to detect and remove the noisy training data, where the “noisiness” is inferred from the amount of gap between the out-of-fold prediction results and the training label. By excluding the training data with a large gap, we were able to construct a “cleaned” training set to train our final models. This label cleaning method considerably improved performance (private leaderboard score from 0.92 to 0.94), making it a main contribution towards winning the challenge.

Data preparation:
We generated tiles for each training dataset using the iafoss-tiling method [2]. We generated two different types of tiles, with the number of tiles being 64 or 36 and the tile height/width being 192 or 256 pixels, respectively, allowing the models to capture a more diverse set of features. Finally, the original ISUP grade group labels were converted by the public binning method [3] to vector format (e.g. ISUP grade group 3 converted to [1, 1, 1, 0, 0]).

Training setup:
For our pipeline, we trained three networks. Network 1 was trained with the original labels and used for cleaning the label noise. Networks 2 and 3 were trained with the cleaned data and used for the final predictions. Networks 1 and 3 were based on EfficientNet-B1 [1] with generalized-mean pooling (GeM pooling) [4] and used the 64 x 192 x 192 tiles as input. Network 2 was based on EfficientNet-B0 with average pooling and used the 36 x 256 x 256 tiles as input. We used the Adam optimizer [5] with a learning rate 0.0003 and Cosine Annealing scheduling with 20-30 epochs.

After training Network 1 with 5 CV folds, we predicted on the out-of-fold (OOF) data. Then, the absolute differences (gaps) between the prediction and the original training label were calculated. If the gap for a sample was higher than a given threshold, the sample was excluded. We used a different threshold for Network 2 and Network 3, resulting in the removal of 5.6% and 14.0% of the training dataset, respectively.

All networks were developed using PyTorch. The following data augmentation procedures were used: flipping, rotating, scaling, color alterations (brightness, and contrast), distortion (grid, optical), Cutout.

Model parameters: 6,937,034 (trainable 6,937,034).

Inference setup:
We used the predictions of Networks 2 and 3. Since the networks were trained on 5 folds, we simply averaged the outputs of the five models to obtain the final prediction. Next, we converted all binnings to ISUP grade groups (e.g. [0.8, 0.7, 0.4, 0.5, 0.2] → 2.6). Finally, we converted the outputs to integers using the following thresholds: [0.5, 1.5, 2.5, 3.5, 4.5] (e.g. 3.4 → 3). We did not optimize these thresholds to avoid overfitting.

Acknowledgements:
I wish to thank my teammate for advice on experimental design.

References:
1. TAN, Mingxing; LE, Quoc. EfficientNet: Rethinking Model Scaling for Convolutional Neural Networks. In: International Conference on Machine Learning. 2019. p. 6105-6114.
2. https://www.kaggle.com/iafoss/panda-16x128x128-tiles
3. https://www.kaggle.com/haqishen/train-efficientnet-b0-w-36-tiles-256-lb0-87
4. WANG, Zhuoqun, et al. Selective Convolutional Features based Generalized-mean Pooling for Fine-grained Image Retrieval. In: 2018 IEEE Visual Communications and Image Processing (VCIP). IEEE, 2018. p. 1-4.
5. Diederik P. Kingma, & Jimmy Lei Ba. (2015) Adam, a Method for Stochastic Optimization, ICLR 2015

Team: rähmä.ai

Joni Juvonen[1], Mikko Tukiainen[1], Antti Karlsson[2]

[1]Silo AI, Turku, 20520, Finland
[2]University of Turku, Turku university hospital, Auria Biobank

Contact: joni.juvonen@silo.ai, mikko.tukiainen@silo.ai, aspkar@utu.fi (corresponding author)

Code and model availability: https://github.com/jpjuvo/PANDA-challenge-raehmae

Abstract:
Our approach was based on the MIL idea by *iafoss*. We augmented the method with our own tile sampling algorithm. We first find the one dimensional skeleton representation of the WSI and then sample data along the skeleton. The method does not require a priori gridding of the WSI, is especially suitable for core needle biopsies because of their elongated shape and produces samples along the medial axis of the biopsy. We then built a classifier and various ordinal regression models based on the *iafoss* model backbone.

Data preparation:
Skeleton sampling was used in all cases of training data generation. In short, we first find the tissue mask with Otsu's thresholding [1]. The tissue mask is then smoothed with standard methods in OpenCV. The smoothed mask is then skeletonized with skimage and the tiles are sampled along the skeleton. Smoothing the mask is crucial to produce a good quality skeleton.

We extracted the tiles at the middle resolution level of the WSI for all of the models. For model 1 we used a maximum of 24 tiles per biopsy, extracted them at 384 x 384 pixels, and resized to 256 x 256. For models 2 and 3 we used a maximum of 36 tiles extracted at 256 x 256 pixels with no resizing. For model 4 we used a maximum of 25 tiles extracted at 299 x 299 pixels with no resizing.

If the biopsies produced more than the maximum number of tiles, we randomly sampled the maximum number of tiles. If the biopsy consisted of many separate parts, each part was sampled with a relative weight based on the length of its corresponding skeleton. We used OpenCV and thresholding based methods to try to avoid sampling the pen annotation marks from the biopsies.

Training setup:
All of the models were based on the ISUP grade group labels and did not use the provided masks in any way. Models 1-3 were all ordinal regression models (predicting ISUP grade groups 0-5) built on top of the approach by *iafoss*. Model 4 was a classification model built on top of the *iafoss* approach (predicting classes 0-5 where 0 means benign). The loss functions used were BCE loss for ordinal regression and CE loss for classification.

The backbone CNN used for feature extraction in all of the models was a pre-trained resnext50_32x4d_ssl model from semi-supervised-ImageNet1K-models [2].

We used 4-fold CV in training all of the models. The number of epochs the models 1, 2, 3 and 4 were trained for were 15, 20, 20 and 20, respectively. All of the models used a flat and anneal learning rate schedule with maximum learning rates of 2e-4, 1e-4, 8e-5 and 7e-4, respectively. We used the “Over9000” optimizer and a batch size of 11. No early stopping was used and only the model with the best validation loss was saved from each training run.

We used data augmentation when training the models. The augmentations were applied uniformly meaning that all of the tiles from the same biopsy were transformed with the same augmentations. The used augmentations were random vertical flips, random zooms, random rotations and random lighting transformations.

Model parameters:
The ordinal regression models had 25,081,157 parameters, all of which were trainable. The classification model had 25,081,670 parameters, all of which were trainable.

Inference setup:
For the inference phase we used an ensemble of models. For models 1 and 2 we chose two versions trained on different CV folds. The models were chosen based on their public leaderboard performance. For models 3 and 4 we chose one fold with the best public leaderboard performance. In total we had 6 models in our ensemble.

To infer the ISUP grade group of a biopsy we used the models in the following way. The results from each ordinal regression model were summed to produce an integer grade. For example (1,0,1,0,0) would be summed to 2. The results from the ordinal regression models and the classification model were averaged with a weighted average. The weights were manually tuned according to how the public leaderboard score was affected by different combinations. The result was rounded to the nearest integer to produce the ISUP grade group for a WSI.

Acknowledgements:
The team warmly acknowledges Auria clinical informatics for providing the computing infrastructure to use for the competition free of charge. The team also thanks Dr. Pekka Taimen and Dr. Eva-Maria Talvitie for interesting discussions. We warmly acknowledge the fellow Kaggler *iafoss* for the original model backbone idea and code on which we built our solution.

References:
1. Otsu, Nobuyuki. "A threshold selection method from gray-level histograms." IEEE transactions on systems, man, and cybernetics 9.1 (1979): 62-66.
2. Facebook Research, https://github.com/facebookresearch/semi-supervised-ImageNet1K-models

Team: Save The Prostate

Rui Guo[1], Chia-Lun Hsieh[2], Igor Zubarev[3] and Habib.S.T.Bukhar[4]

[1]University of Michigan, Ann Arbor, Michigan, 48104, USA
[2]No. 7, Daxing Street, 13, Datong Lane, Beitou District, Taipei City, Taiwan
[3]Independent researcher, Tula, Russia
[4]Janelia Research Campus, Ashburn, 20148, USA

Contact: guorui@umich.edu, rayxie0329@gmail.com, zubarev.ia@gmail.com, bukharih@janelia.hhmi.org

Code and model availability: https://github.com/DrHB/PANDA-2nd-place-solution

Abstract:
Deep learning methods have shown promising results in diagnosing prostate cancer. However, due to the various input shapes and giga-pixel resolution of WSIs, traditional training methods require extensive and costly computational resources and specific CNN architectures. Our team has utilized a novel tile-based method to address these issues, which requires less computational resources while maintaining state-of-the-art performance. Our approach includes a combination of three different methods: (1) MIL-based CNN with attention-selected high-resolution input, (2) MIL-based CNN with Squeeze-and-Excite Module across all tiles, (3) Deep CNN with rectangular input images recomposed from tiles. Additionally, we successfully tackled label noise in training data by utilizing robust loss functions and pseudo labels.

Data preparation:
To reduce image dimensions and fix the input size, we first cropped WSIs into tiles. Then we summed the pixel values in these tiles and selected those with lower summed values (i.e. darker content). Two of our models used separate tiles as input and the third one concatenated these tiles into a large image. Additionally, we removed slides with pen marks and with inconsistencies between the slide-level labels and masks.

Training setup:
In total, we trained four models, which we can divide into three categories: (1) MIL-based CNN with attention-selected high-resolution input, (2) MIL-based CNN with SE Module across all tiles, (3) Deep CNN with recomposed rectangular images from tiles. Figure A3 shows the structures of these networks. We used weights pretrained on ImageNet (standard or noisy student [1]) and applied augmentations at both the WSI and tile levels for all models. The WSI was randomly cropped to 95% of the original size and rotated in the range from -5 degrees to 5 degrees. Tile-level augmentation included random brightness and contrast, horizontal and vertical flips, random rotation of -10 to +10 degrees, and cutout.

MIL-based CNN with attention-selected high-resolution input

The model is trained in two stages. This model takes separate tiles as input and uses attention pooling [2] to gather information from tiles in the same WSI. In the first stage, models were trained on the middle resolution level WSIs, with 48 tiles having dimensions of 192 x 192 pixels selected based on color information. The model used a Se_ResNeXt50_32x4d [3] backbone and was trained with three losses: MSE loss for regression, CrossEntropy for classification, and tile-level classification. The Adam optimizer [4], with a learning rate of 5e-4 and a batch size of 6, was used for model fitting. The learning rate was scaled by 0.2 at the 20th, 35th and 45th epoch. The model with the best QWK on the validation set was chosen.

The models were finetuned on 2x the intermediate resolution in the second stage, with 36 tiles selected by precalculated attention values from stage one. To reduce label noise, the label for MSE regression was a weighted sum of ground truth and pseudo labels from the first stage. The models used EfficientNet-b0 [5] as the backbone and weights obtained from stage one as initialization. The Adam optimizer with a learning rate of 2e-4 and 20 epochs of cosine decay was used to fit the models.

MIL-based CNN with Squeeze-and-Excite Module across all tiles

Two models were trained using this approach: ResNeXt50 and Resnet34. For ResNeXt50 we split WSIs into 49 tiles of size 224 x 224 pixels. These tiles were passed through the backbone to produce feature vectors. The global SE module was then applied across tiles from the same WSI. After the SE module, feature vectors from the same WSI were pooled into one feature vector. Because Batch Normalization works poorly with very small batch sizes, we replaced all Batch Normalization layers with Group Normalization layers, which allowed this particular model to successfully converge with batch sizes as low as 1. Additionally, Weight Standardization [6] was added to each Convolutional layer to accelerate micro-batch training. The last layer contained a single output neuron with a sigmoid range function. We used the L1 Smooth loss function. ResNeXt50 was trained using a reduction on plateau scheduler with an Initial learning rate of 4e-4, which was scaled by a factor of 0.5 every seven epochs without improvement in the validation metric.

Training of Resnet34 was done in two stages: first trained with 49 tiles per WSI and then finetuned with 81 tiles. The model had a similar structure as ResNext50 with Se Block followed by two pooling layers. The last layer contained two output layers, one for classification and one for regression with a sigmoid range. The training was done using the two-loss function, Cross-Entropy, and Mean Square Error. Both models were trained end-to-end with a Radam optimizer. We used a one cycle schedule with a learning rate of 1e-3 for 80 (stage 1) and 40 (stage 2) epochs.

Recomposed image based approach

This model used large rectangular images composed from 144 tiles at 128 x 128 pixels. The background in each tile was cropped out and the foreground was resized to 128 x 128. The EfficientNetB3 [5] network with noisy-student pre-trained weights [1] was used as a model backbone, followed by the GeM pooling layer and a final single regression head. The model was

trained using two different loss functions: Huber loss for Radboud data and mean square error for Karolinska data. The model was trained on a single TPU with a 5e-4 initial learning rate using the Adam optimizer and one cycle policy. The training was stopped when Karolinska's loss stopped declining.

Model parameters:
Model(1), Backbone: EfficientNet-b0 4.6M
Model(1), Backbone: SeResNeXt50_32x4d 28.6M
Model(2), Backbone: ResNet 34 21.8 M
Model(2), Backbone: ResNeXt50 25M
Model(3), Backbone: EfficientNet-b3 12M

Inference setup:
***MIL-based CNN with attention selected tiles**.* The model was first run at the intermediate resolution level, taking every tile as input and calculating attention values for each tile. Then the top 36 tiles were selected, and we cropped tiles at the same location in the highest resolution image and downsampled them by a factor of two. Finally, the model was run on these higher resolution tiles, and we took the outputs from the regression head.
***MIL-based CNN with Squeeze-and-Excite Module across all tiles**.* We extracted images at the intermediate resolution level and split the tissue into tiles. We divided the biopsy into 81 (Resnet) and 49 (ResNeXt50) tiles, and computed raw predictions from the regression head.
***Re-composed image based approach**.* We extracted 144 tiles with 128 x 128 pixels from the intermediate resolution level and concatenated them into a large image. Similar to training, the backgrounds in these tiles were cropped out and the foregrounds were resized. Finally, we took the raw prediction from regression.
Ensemble and Rounding. At the end all the predictions were averaged and rounded to produce the final ISUP grade group.

Acknowledgements:
We would like to thank Kaggle users *lafoss, haqishen* and *dannellyz* for publicly sharing valuable ideas.

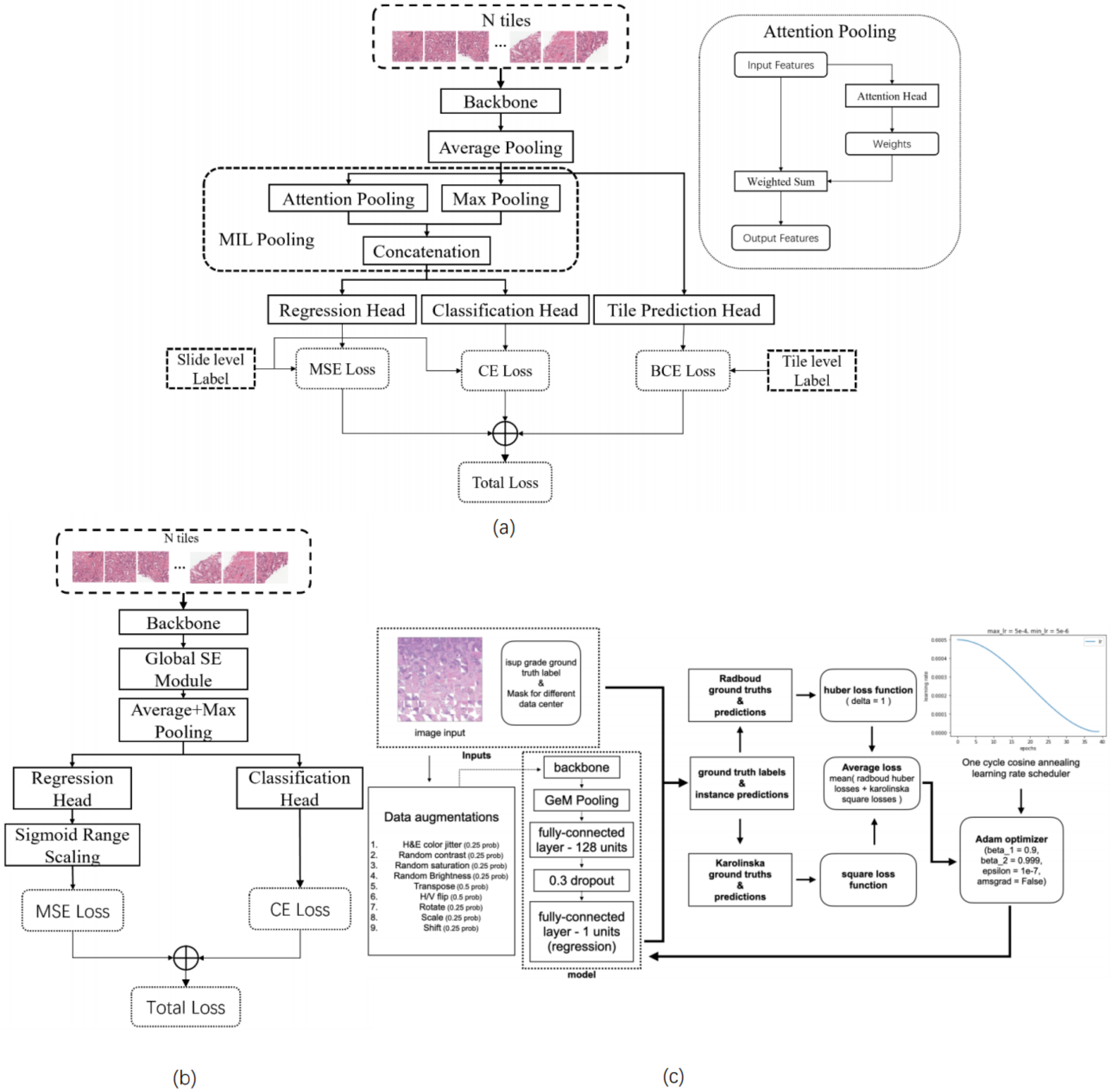

Figure A3: Overview of the solution of *Save The Prostate*. (a) MIL-based CNN with attention-selected high-resolution input. The model takes N separate tiles as input and uses a MIL pooling to fuse feature vectors from N tiles in the same WSI into one feature vector. Attention values calculated in attention pooling are used to select tiles in higher resolution. (b) MIL based CNN with Squeeze-and-Excite Module across all tiles. This model takes N separate tiles as input. The global Squeeze-and-Excite module will operate on all tiles from the same WSI. All tiles from the same WSI will share the channel attention in SE operation. (c) Deep CNN with re-composed rectangular images from tiles. This model used re-composed rectangular images as input. The large images are generated by concatenating small tiles selected from WSIs. Difference losses are applied to data from different centers.

References:

1. Oizhe Xie, Minh-Thang Luong, Eduard Hovy, and Quoc V. Le. Self-training with noisy student improves imagenet classification. In Proceedings of the IEEE/CVF Conference on Computer Vision and Pattern Recognition (CVPR), June 2020.
2. Maximilian Ilse, Jakub Tomczak, and Max Welling. Attention-based deep multiple instance learning. volume 80 of Proceedings of Machine Learning Research, pages 2127–2136, 2018. PMLR.
3. J. Hu, L. Shen, & G. Sun (2018). Squeeze-and-Excitation Networks. In 2018 IEEE/CVF Conference on Computer Vision and Pattern Recognition (pp. 7132-7141).
4. Diederik P. Kingma, & Jimmy Lei Ba. (2015) Adam, a Method for Stochastic Optimization, ICLR 2015
5. Mingxing Tan and Quoc V Le. Efficientnet: Rethinking model scaling for convolutional neural networks. arXiv preprint arXiv:1905.11946, 2019.
6. Siyuan Qiao, Huiyu Wang, Chenxi Liu, Wei Shen, and Alan Yuille. Micro-batch training with batch-channel normalization and weight standardization, arXiv preprint arXiv:1903.10520, 2019.

Team: UCLA Computational Diagnostics Lab

Wenyuan Li[1], Jiayun Li[1], William Speier[1], Corey Arnold[1]

[1]Computational Diagnostics Lab, University of California, Los Angeles, Los Angeles, 90024, USA

Contact: liwenyuan.zju@gmail.com, jiayunli@ucla.edu, speier@ucla.edu, cwarnold@ucla.edu.

Code and model availability: Code and model not shared.

Abstract:
We developed an algorithm based on an attention-based multi-resolution model ensembled with LGBM [5] and XGBoost [6]. The algorithm contains a two-stage attention-based MIL model that uses weakly supervised region of interest (ROI) detection. Our model was trained on tiles extracted from WSIs on multiple resolutions, with the lower resolution used to identify suspicious regions, which were further examined at higher resolution. To make the model more robust, we ensembled the MIL model with LGBM and XGBoost models, whose feature extractors were trained to predict the primary and secondary Gleason grades.

Data preparation:
We first performed data cleaning including removal of pen marks and suspicious slides (blank slides and slides with no cancerous tissue indicated by the mask but with ISUP grade group greater than 0), as well as detection of duplicates. We ensured that each set of duplicates was split into the same fold during CV. After data cleaning, we performed data pre-processing using our previously developed tiling algorithm [1]. Specifically, we created a mask of the tissue on the slide by setting a threshold for the average intensity since the majority of the background is white. Once a tissue mask was found, it was smoothed using morphological closing. The skeleton of the smoothed mask was then found, and branches were removed by finding the endpoints with the maximum geodesic distance. The midline was then partitioned based on the tile size and overlap. A perpendicular line was drawn at each of the locations until it intersected with the mask boundary. A tile size of 256 x 256 pixels was used with an overlap of s = 25% in this challenge. Finally, we calculated the blue ratio for each tile, and the top 36 ranked tiles were selected. The selected tiles were concatenated to feed the MIL, LGBM, and XGBoost models. In our attention-based MIL model, each extracted tile was considered as an instance and each slide was modeled as a bag of instances.

Training setup:
Our final model was an ensemble of three components: attention-based MIL, LGBM and XGBoost. Our attention-based MIL model [2] consisted of two stages, which operated on two resolutions. ResNeXt50[3] was used as the backbone for both stages. Adaptive 2D average pooling and 2D convolution were applied after the last convolutional layer of the ResNeXt50 to produce a $k \times 512 \times 4 \times 4$ feature map for each slide, which was then flattened and projected to $k \times 512$ instance-level feature vectors. k denotes the number of extracted tiles,

and was set to 36 for both stage models. Feature vectors were aggregated by soft attention weights generated from the attention module (i.e., multi-layer perceptron) to form a bag-level representation for grade classification.

The input tile size for the first stage was 256 x 256 pixels. To select informative regions for the second stage, from each tile with top 50% highest attention weights obtained from the first stage model, we extracted two tiles of size 512 x 512 at the highest resolution around the center of 64 x 64 sub-regions at intermediate resolution with highest blue ratio values. The 512 x 512 tiles were then down-sampled to 256 x 256 and forwarded to the second stage model. The other two components, i.e. LGBM and XGBoost were fairly simple compared to MIL. We employed ResNeXt50 as an image feature extractor on the intermediate resolution. To diversify the model, the end task we used here was to predict the Gleason score. We formulated the primary and secondary Gleason grade prediction as a multi-task learning task. We extracted the image features for each WSI using ResNeXt50 and used these fixed feature vectors to train LGBM and XGBoost separately for the final ISUP grade group prediction. To combine the predictions from these three models, we trained linear regression to output a continuous number from 0 to 5.

For training, we used the Adam optimizer [4] with an initial learning rate of 0.0003 and the cosine annealing learning rate scheduler. The number of training epochs was 30 and the batch size was 6. Models were trained and evaluated using 4-fold CV.

Model parameters:
MIL: 27,268,914 (trainable 27,268,914).
LGBM: 25,081,166 (trainable 25,081,157).
XGBoost: 25,081,180 (trainable 25,081,157).
Total: 77,431,260, (trainable 77,431,228).

Inference setup:
We used the ensemble model for ISUP grade group prediction. Specifically, we applied the first stage attention MIL model on the intermediate resolution tiles for grade prediction and suspicious region localization. Then selected tiles were examined at a higher resolution by our second stage model. Final predictions were obtained by averaging the predictions from both stages. Similarly, LGBM and XGBoost were used to predict an ISUP grade group simultaneously. A pre-trained linear regression model was used to aggregate the predictions from these three models. It mapped the final prediction to a continuous scale from 0 to 5. To determine the proper thresholds for the final ISUP grade group outcome, we tried to optimize a differential equation where we used the thresholds as variables and QWK as the objective score. The threshold finetuning step could increase the final leaderboard score by up to 1%.

Acknowledgements:
We would like to acknowledge support from the NIH/NCI R21CA220352 grant program. This research was also enabled in part by GPUs donated by NVIDIA Corporation. We would also like to thank other competitors for their helpful discussions and kernels. These include kernels for

data cleaning by *akensert, Zac Dannelly,* and *Appian*, and preprocessing by *rftexas*, network architectures by *lafoss,* and training details by *haiqishen* and *abhishek*.

References:

1. Speier, W., Li, J., Li, W., Sarma, K., & Arnold, C. (2020). Image-based patch selection for deep learning to improve automated Gleason grading in histopathological slides. bioRxiv.
2. Li J, Li W, Gertych A, Knudsen BS, Speier W, Arnold CW. An attention-based multi-resolution model for prostate whole slide image classification and localization. arXiv preprint arXiv:1905.13208. 2019 May 30.
3. Xie S, Girshick R, Dollár P, Tu Z, He K. Aggregated residual transformations for deep neural networks. In Proceedings of the IEEE conference on computer vision and pattern recognition 2017 (pp. 1492-1500).
4. Diederik P. Kingma, & Jimmy Lei Ba. (2015) Adam, a Method for Stochastic Optimization, ICLR 2015
5. Ke, G., Meng, Q., Finley, T., Wang, T., Chen, W., Ma, W., Ye, Q. and Liu, T.Y., 2017. Lightgbm: A highly efficient gradient boosting decision tree. In Advances in neural information processing systems (pp. 3146-3154).
6. Chen, T. and Guestrin, C., 2016, August. Xgboost: A scalable tree boosting system. In Proceedings of the 22nd acm sigkdd international conference on knowledge discovery and data mining (pp. 785-794).

Team: vanda

Kyungdoc Kim[1], Byeonguk Bae[1], Yeong Won Kim[1], Hong-Seok Lee[1], Jeonghyuk Park[1]

[1]VUNO Inc., Seoul, 06536, Republic of Korea

Contact: kyungdoc.kim@vuno.co

Code and model availability: Code and model not shared.

Abstract:
We developed an algorithm based on the ResNext50 [1] model. Our model was trained on 64 tiles of 256 x 256 x 3 pixels extracted from the intermediate resolution level of the WSIs. Since the ISUP grade group is defined on an ordinal scale, we adopted the mean-variance loss (Mvloss) [2]. We split the dataset into four folds such that consecutive tissue sections of the same sample were not divided into different folds. Various image augmentations were applied to the tiles. We also adopted unsupervised data augmentation (UDA) for consistency training [3], and it improved the final performance especially on the Radboud data. We used eight different test-time-augmentations.

Data preparation:
All WSIs of the training dataset were used at the intermediate resolution, and tiles were generated using the public notebook by *akensert*. We used a tile size of 256 x 256 x 3 pixels, and applied zero padding to tiles located at the borders of WSIs. Meanwhile, suspicious WSIs with inconsistent labels or no mask were removed from the training dataset. Serial WSIs representing consecutive tissue sections were grouped using image hash, and WSIs from the same group were not divided into different folds. Most of the serial WSIs were derived from the Radboud data.

Training setup:
Our model was based on the ResNext50 model pre-trained on ImageNet proposed by *iafoss*. We followed the base model, and extended the model by adding separated additional classification heads for Gleason scores or merged one with the ISUP grade group. However, the base model itself showed the best performance based on exhaustive experiments.

We used Mvloss to take into account the ordinal relationships between ISUP grade groups. Mvloss is the weighted sum of the cross-entropy loss, and the L2 loss of the expected value of the probability and the variance value of the probability. We set 0.2 and 0.05 as the weight of the L2 loss and the variance value, respectively. Meanwhile, UDA was adopted to improve the generalization of the models [3]. During the training, each batch was duplicated and processed with different augmentations. As an additional loss, KL divergence was computed between logits from the two batches. The optimizer minimized both Mvloss and the KL divergence simultaneously.

Due to limited GPU memory, the batch size was set to 6 with UDA or 12 without UDA on four GPUs. In order to increase the batch size, we trained the model on four GPUs with half floating point precision. We used the Over9000 optimizer with cosine annealing. For data augmentation, we used image flipping, random rotation by 90 degrees, random gamma adjustment, ShiftScaleRotate, and RandomBrightnessContrast in Albumentations. The final models used in the ensemble have slightly different hyperparameters, but most of them were trained with a learning rate of 0.0001 and 60 epochs.

Model parameters:
We used an ensemble of 12 models, with each having 25,081,670 trainable parameters.

Inference setup:
The final models for the inference were the top three with highest QWK from each fold. Since we split the dataset into four folds, this resulted into an ensemble of 12 models. For the test data, we extracted 64 tiles per WSI, adding white tiles when the number of tiles was insufficient. Since test-time-augmentation was applied to the tiles, the inference GPU used in Kaggle managed to run the inference for each WSI. All predictions were averaged and then rounded to determine the final ISUP grade group.

Acknowledgements:
The authors would like to thank PANDA challenge organizers and all participants. Especially, thanks to *iafoss, akensert*, and *appian* for their public notebooks.

References:
1. Xie S, Girshick R, Dollár P, Tu Z, He K. Aggregated residual transformations for deep neural networks. In Proceedings of the IEEE conference on computer vision and pattern recognition 2017 (pp. 1492-1500).
2. Pan, Hongyu, et al. Mean-variance loss for deep age estimation from a face. Proceedings of the IEEE Conference on Computer Vision and Pattern Recognition. 2018.
3. Xie, Qizhe, et al. Unsupervised data augmentation for consistency training. arXiv preprint arXiv:1904.12848, 2019.
