# Supplementary material for: Artificial intelligence for diagnosis and Gleason grading of prostate cancer: the PANDA challenge
Source: Nat Med. 2022 Jan 13;28(1):154–63. doi: 10.1038/s41591-021-01620-2 (PMC8799467; doi:10.1038/s41591-021-01620-2)
Supplement: Supplementary file 2 — Reporting Summary [file 41591_2021_1620_MOESM2_ESM.pdf]

nature research

Corresponding author(s): Wouter Bulten, Kimmo Kartasalo, Po-Hsuan Cameron Chen

Last updated by author(s): Oct 19, 2021

Reporting Summary

Nature Research wishes to improve the reproducibility of the work that we publish. This form provides structure for consistency and transparency in reporting. For further information on Nature Research policies, see our Editorial Policies and the Editorial Policy Checklist.

Statistics

For all statistical analyses, confirm that the following items are present in the figure legend, table legend, main text, or Methods section.

n/a	Confirmed	
☐	☒	The exact sample size (*n*) for each experimental group/condition, given as a discrete number and unit of measurement
☐	☒	A statement on whether measurements were taken from distinct samples or whether the same sample was measured repeatedly
☐	☒	The statistical test(s) used AND whether they are one- or two-sided<br>*Only common tests should be described solely by name; describe more complex techniques in the Methods section.*
☒	☐	A description of all covariates tested
☒	☐	A description of any assumptions or corrections, such as tests of normality and adjustment for multiple comparisons
☐	☒	A full description of the statistical parameters including central tendency (e.g. means) or other basic estimates (e.g. regression coefficient) AND variation (e.g. standard deviation) or associated estimates of uncertainty (e.g. confidence intervals)
☒	☐	For null hypothesis testing, the test statistic (e.g. F, t, r) with confidence intervals, effect sizes, degrees of freedom and P value noted<br>*Give P values as exact values whenever suitable.*
☒	☐	For Bayesian analysis, information on the choice of priors and Markov chain Monte Carlo settings
☒	☐	For hierarchical and complex designs, identification of the appropriate level for tests and full reporting of outcomes
☐	☒	Estimates of effect sizes (e.g. Cohen's *d*, Pearson's *r*), indicating how they were calculated

Our web collection on statistics for biologists contains articles on many of the points above.

Software and code

Policy information about availability of computer code

Data collection	After scanning the slides, data was converted and exported using the open source ASAP software (version 1.9, https://github.com/computationalpathologygroup/ASAP). Instructions on how to use the data are included in the following repository: https://github.com/DIAGNijmegen/panda-challenge
Data analysis	Analysis was performed using Python (version 3.8) in combination with the following software packages: scipy (1.5.4), pandas (1.1.4), mlxtend (0.18.0), numpy (1.19.4), scikit-learn (0.23.2), matplotlib (3.3.2), jupyterlab (2.2.9) and notebook (6.1.5).<br><br>Code for the analysis of algorithm performance is made publicly available through https://github.com/DIAGNijmegen/panda-challenge/<br><br>The Docker image that all the algorithms were based on is available online at https://github.com/Kaggle/docker-python. On the Puhti GPU cluster, the Docker images were automatically converted for use with Singularity (version 3.8.3). Details on the availability of specific models and the code of the contributed algorithms can be found in the supplementary algorithm descriptions.

For manuscripts utilizing custom algorithms or software that are central to the research but not yet described in published literature, software must be made available to editors and reviewers. We strongly encourage code deposition in a community repository (e.g. GitHub). See the Nature Research guidelines for submitting code & software for further information.

Data

Policy information about availability of data

All manuscripts must include a data availability statement. This statement should provide the following information, where applicable:

- Accession codes, unique identifiers, or web links for publicly available datasets
- A list of figures that have associated raw data
- A description of any restrictions on data availability

The full development set, from here on named the PANDA Challenge dataset, of 10,616 digitized de-identified H&E stained prostate biopsies (383GB) will be made publicly available for further research. The data can be used under a Creative Commons BY-SA-NC 4.0 license. To adhere to the "Attribution" part of the license, we ask anyone who uses the data to cite the corresponding paper. The most up-to-date information regarding the dataset will be published on the challenge website at https://panda.grand-challenge.org/

Field-specific reporting

Please select the one below that is the best fit for your research. If you are not sure, read the appropriate sections before making your selection.

☒ Life sciences ☐ Behavioural & social sciences ☐ Ecological, evolutionary & environmental sciences

For a reference copy of the document with all sections, see nature.com/documents/nr-reporting-summary-flat.pdf

Life sciences study design

All studies must disclose on these points even when the disclosure is negative.

Sample size	No formal sample size calculation was performed. We collected as many samples as possible, considering samples that were readily available in digital format across the institutions involved in this study, and taking into account that the dataset size needed to remain feasible to download (at < 500 GB) for competition participants. For the validation sets, we combined the cohorts of Bulten et al. LO 2020, Ström et al. LO 2020, and Nagpal et al. JAMA Onc 2020, resulting in a sample size far surpassing earlier work and capturing a wide range of the morphological heterogeneity present in prostate needle core biopsies.
Data exclusions	During the data collection for the development, tuning, and validation sets, a total of 111 biopsies were excluded due to risk of information leakage (e.g. pathologist pen markings visible on the tissue), poor staining, or image quality issues. While establishing the reference standard, 65 biopsies were excluded due to a lack of consensus among the pathologists providing the reference standard. More details on exclusion criteria are displayed in the supplementary appendix.<br><br>No data were excluded from the analysis. All algorithms selected for the study among the competition participants were included in the analysis. All selected algorithms produced results for all included cases.
Replication	All training data is made publicly available, which allows for replication of the algorithms. For several algorithms, the code has also been made open source. Details on the availability of specific models and the code of the contributed algorithms can be found in the supplementary algorithm descriptions. Code for the analysis of algorithm performance is made publicly available through https://github.com/DIAGNijmegen/panda-challenge/<br><br>The authors independently reproduced all algorithms contributed by the challenge participants. All details on this process are described in the main text and supplementary materials.
Randomization	Samples were per data provider randomly allocated into development (N = 10,616), tuning (N = 393) and internal validation (N = 545) sets, stratified by Gleason score. All samples from a given patient were assigned to the same set. External validation sets were collected fully independently from the development, tuning and internal validation data and were thus not part of the randomization process.
Blinding	The challenge organizers had access to all data in the study. The algorithm developers had no access to the data used for the validation of the algorithms. Algorithms were independently applied to the validation sets by the challenge organizers, without the involvement of the original developers. Running the algorithms on the validation sets was done programmatically without manual intervention or any algorithmic modifications by the challenge organizers. The algorithms' output was fixed and stored in a repository before the statistical analysis was performed.

Reporting for specific materials, systems and methods

We require information from authors about some types of materials, experimental systems and methods used in many studies. Here, indicate whether each material, system or method listed is relevant to your study. If you are not sure if a list item applies to your research, read the appropriate section before selecting a response.

Materials & experimental systems

n/a	Involved in the study
☒	☐ Antibodies
☒	☐ Eukaryotic cell lines
☒	☐ Palaeontology and archaeology
☒	☐ Animals and other organisms
☐	☒ Human research participants
☒	☐ Clinical data
☒	☐ Dual use research of concern

Methods

n/a	Involved in the study
☒	☐ ChIP-seq
☒	☐ Flow cytometry
☒	☐ MRI-based neuroimaging

Human research participants

Policy information about studies involving human research participants

Population characteristics

All cases were retrospectively collected histological H&E stained tissue sections of prostate biopsy specimens, acquired from men who underwent a biopsy procedure due to suspicion of prostate cancer in one of the six institutions included in the study. The patient age distribution was as follows. EU internal validation set (Karolinska Institutet): <54 y (3.7%), 55-59 y (11.0%), 60-64 y (23.2%), 65-69 y (58.5%), >= 70 y (3.7%); EU external validation set (Karolinska University Hospital): <54 y (9.5%), 55-59 y (13.7%), 60-64 y (16.4%), 65-69 y (20.5%), >= 70 y (39.7%); US external validation set medical laboratory 1: <65 y (44.2%), >= 65 y (51.6%), not available (4.2%); US external validation set medical laboratory 2: <65 y (41.0%), >= 65 y (57.5%), not available (1.6%). Further details are provided in the supplementary appendix.

Recruitment

Cases were retrospectively included at random, sourced through three independent studies, across six sites. For the Radboud data, we retrieved all pathology reports dated between Jan 1, 2012, and Dec 31, 2017, for patients who underwent a prostate biopsy owing to a suspicion of prostate cancer. Patients were randomly sampled based on the highest reported Gleason score mentioned in each report. Additionally, a set of reports was sampled which only mentioned benign biopsies. The data from Karolinska comes from the Stockholm-3 diagnostic trial that was conducted between May 28, 2012 and Dec 30, 2014, (ISRCTN84445406). It was a prostate cancer screening-by-invitation trial of men aged 50–69 years living in Stockholm, Sweden. The purpose of the trial was to compare prostate specific antigen (PSA) to the Stockholm-3 model (S3M) for predicting the presence of cancer, and the criterion for referral to biopsy was either PSA above 3 ng/ml or a S3M probability of 10% or higher. A random sample from the biopsies included in the trial was taken, stratified on patient and the reported Gleason score to avoid including too many of the prevalent benign and low grade diseases. The US external validation set consisted of retrospective cases from three different sources. Briefly, cases were obtained from two medical laboratories and one tertiary teaching hospital. All tumor-containing cases available from the tertiary teaching hospital from 2005-2007 were included, and a fraction of the benign biopsies available were randomly sampled for inclusion. From the medical laboratories, all available ISUP grade group 4-5 cases were included in the study, and remaining benign and ISUP grade group 1-3 cases were randomly sampled for inclusion. The EU external validation set comprised biopsy cores assessed by L.E. at the Karolinska University Hospital during 2018. The set included all positive biopsy cores from all men diagnosed with an ISUP grade group 2, 3, 4, or 5 cancer as well as from a random selection of men diagnosed with ISUP grade group 1 cancer during that time period. In addition, the set included all cores from a random selection of men with only benign biopsies.

Ethics oversight

The study was approved by the institutional review board of Radboud University Medical Center (IRB 2016–2275), Stockholm regional ethics committee (permits 2012/572-31/1, 2012/438-31/3, and 2018/845-32), and Advarra (Columbia, MD; Pro00038251).

Note that full information on the approval of the study protocol must also be provided in the manuscript.
